# Prions activate a p38 MAPK synaptotoxic signaling pathway

**DOI:** 10.1371/journal.ppat.1007283

**Published:** 2018-09-20

**Authors:** Cheng Fang, Bei Wu, Nhat T. T. Le, Thibaut Imberdis, Robert C. C. Mercer, David A. Harris

**Affiliations:** Department of Biochemistry, Boston University School of Medicine, Boston MA, United States of America; Istituto Superiore di Sanità, ITALY

## Abstract

Synaptic degeneration is one of the earliest pathological correlates of prion disease, and it is a major determinant of the progression of clinical symptoms. However, the cellular and molecular mechanisms underlying prion synaptotoxicity are poorly understood. Previously, we described an experimental system in which treatment of cultured hippocampal neurons with purified PrP^Sc^, the infectious form of the prion protein, induces rapid retraction of dendritic spines, an effect that is entirely dependent on expression of endogenous PrP^C^ by the target neurons. Here, we use this system to dissect pharmacologically the underlying cellular and molecular mechanisms. We show that PrP^Sc^ initiates a stepwise synaptotoxic signaling cascade that includes activation of NMDA receptors, calcium influx, stimulation of p38 MAPK and several downstream kinases, and collapse of the actin cytoskeleton within dendritic spines. Synaptic degeneration is restricted to excitatory synapses, spares presynaptic structures, and results in decrements in functional synaptic transmission. Pharmacological inhibition of any one of the steps in the signaling cascade, as well as expression of a dominant-negative form of p38 MAPK, block PrP^Sc^-induced spine degeneration. Moreover, p38 MAPK inhibitors actually reverse the degenerative process after it has already begun. We also show that, while PrP^C^ mediates the synaptotoxic effects of both PrP^Sc^ and the Alzheimer’s Aβ peptide in this system, the two species activate distinct signaling pathways. Taken together, our results provide powerful insights into the biology of prion neurotoxicity, they identify new, druggable therapeutic targets, and they allow comparison of prion synaptotoxic pathways with those involved in other neurodegenerative diseases.

## Author summary

Prion diseases are a group of fatal neurodegenerative disorders that includes Creutzfeldt-Jakob disease and kuru in humans, and bovine spongiform encephalopathy in cattle. The infectious agent, or prion, that transmits these diseases is a naked protein molecule, the prion protein (PrP), which is an altered form of a normal, cellular protein. Although a great deal is known about how prions propagate themselves and transmit infection, the process by which they actually cause neurons to degenerate has remained mysterious. Here, we have used a specialized neuronal culture system to dissect the cellular and molecular mechanisms by which prions damage synapses, the structures that connect nerve cells and that play a crucial role in learning, memory, and neurological disease. Our results define a stepwise molecular pathway underlying prion synaptic toxicity, which involves activation of glutamate neurotransmitter receptors, influx of calcium ions into the neuron, and stimulation of specific mitogen-activated protein kinases, which attach phosphate groups to proteins to regulate their activity. We demonstrate that specific drugs, as well as a dominant-negative kinase mutant, block these steps and thereby prevent the synaptic degeneration produced by prions. Our results provide new insights into the pathogenesis of prion diseases, they uncover new drug targets for treating these diseases, and they allow us to compare prion diseases to other, more common neurodegenerative disorders like Alzheimer’s disease.

## Introduction

Prion diseases are a group of fatal, infectious neurodegenerative diseases affecting humans and animals. The infectious agent, or prion, is composed of PrP^Sc^, a conformationally altered form of a normal, cell-surface glycoprotein designated PrP^C^. Prions propagate themselves by a highly specific templating process in which PrP^Sc^ molecules impose their unique, β-sheet-rich conformations on endogenous PrP^C^ substrate molecules [1–4]. Consistent with this model, PrP knockout mice, in which PrP^C^ expression is absent, are completely resistant to prion infection [5, 6]. Moreover, these mice do not display symptoms of a prion disease [7], indicating that the disease phenotype is due primarily to a gain-of-function attributable to PrP^Sc^ or a related toxic species, rather than to a loss of the normal function of PrP^C^. Therefore, it is important to understand the molecular mechanism of PrP^Sc^ neurotoxicity.

Strikingly, the question of prion neurotoxicity has received relatively little attention in the field, in comparison to the extensive body of research that has been published on prion infectivity, propagation, and transmission. An important clue to the underlying mechanism is the observation that neurons that do not express endogenous PrP^C^ are relatively resistant to the toxic effects of exogenously supplied PrP^Sc^ [8, 9]. This result suggests that a critical neurotoxic signal is generated as part of the process by which endogenous cell-surface PrP^C^ is converted into PrP^Sc^, and in the absence of PrP^C^, this signal is not produced. Consistent with a role for PrP^C^ as a neurotoxic mediator, there is evidence that prion disease progression in mice is characterized by two, mechanistically distinct phases: rapid accumulation of PrP^Sc^ and infectivity, followed by slower development of neuropathology and clinical symptoms over a time course that is inversely related to expression levels of PrP^C^ [10, 11]. Although there are a number of studies suggesting signal-transducing activities for cell-surface PrP^C^ [reviewed by 12], the pathways by which its interaction with PrP^Sc^ produces neurotoxic signals remain mysterious.

Synaptic loss is a common theme in many neurodegenerative disorders [13, 14]. In prion diseases, neuropathological and *in vivo* imaging studies in infected mice suggest that synaptic degeneration begins very early in the disease process, predating other pathological changes, and eventually contributing to the development of clinical symptoms [15–22]. However, there is very little mechanistic understanding of this process, due largely to the absence of suitable cell culture models amenable to experimental manipulation.

To address this gap, we previously established a novel neuronal culture model, using which we showed that PrP^Sc^ induces rapid retraction of spines on the dendrites of hippocampal neurons [23]. Importantly, this effect is entirely dependent on expression of endogenous PrP^C^ by the neurons, consistent with the previously demonstrated role of PrP^C^ as an essential transducer of PrP^Sc^ toxicity. Dendritic spines are the contact sites for most excitatory synapses in the brain, and they undergo constant morphological remodeling during development, learning, and memory formation [24, 25]. Therefore, spines are an important locus for the pathogenesis of neurological diseases, particularly those involving symptoms of dementia.

Here, we have used cultured hippocampal neurons to dissect, using specific pharmacological inhibitors as well a dominant-negative kinase mutant, the mechanism of PrP^Sc^-induced synaptotoxicity. Our data establish a synaptotoxic signaling pathway involving, in stepwise sequence, activation of NMDA and AMPA receptors, calcium influx, stimulation of p38 mitogen-activated protein kinase (MAPK), and depolymerization of actin filaments in dendritic spines. Blocking any one of these steps prevented dendritic spine retraction in response to PrP^Sc^, and could, in some cases, even restore normal morphology to spines that had already degenerated. Taken together, our results provide powerful insights into the biology of prion neurotoxicity, they identify new, druggable therapeutic targets, and they allow comparison of the synaptotoxic pathways underlying prion diseases with those responsible for other neurodegenerative disorders like Alzheimer’s disease.

## Results

### PrP^Sc^ causes functional changes in synaptic transmission

Previously, we showed that treatment of cultured hippocampal neurons for 24 hrs with purified PrP^Sc^, but not control preparations, induced a dramatic retraction of dendritic spines, an effect that was entirely dependent on expression of endogenous PrP^C^ by the neurons [23].

**Fig 1:**
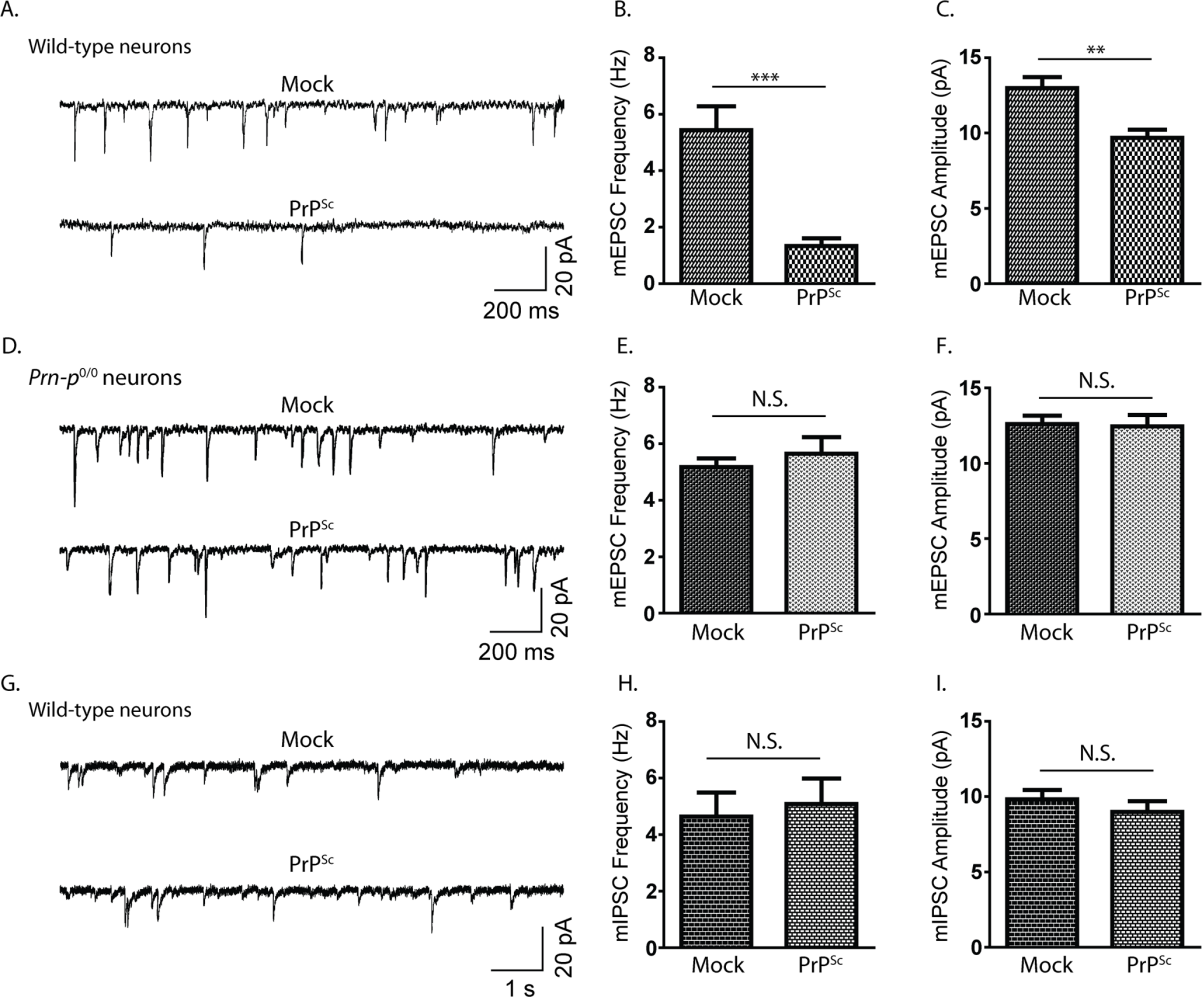
PrP^Sc^ causes a reduction in spontaneous synaptic neurotransmitter release. Hippocampal neurons from WT mice (A, G) or *Prn-p*^0/0^ mice (D) were treated for 24 hrs with either mock-purified material or purified PrP^Sc^. Whole-cell patch clamping was used to record mEPSCs (A, D) and mIPSCs (G), the frequencies and amplitudes of which were quantitated (B,C, E, F, H, I). N = 15 cells from 3 independent experiments. ***p<0.001, **p<0.01, by Student’s t-test; N.S., not significantly different.

Before embarking on pharmacological studies, we undertook several experiments to characterize the synaptotoxic effects of PrP^Sc^ in neuronal culture, and to gain further insight into the underlying cellular mechanisms. First, we wondered whether the dramatic changes in dendritic spine morphology caused by PrP^Sc^ were accompanied by alterations in the efficiency of synaptic transmission. To test this possibility, we used patch clamp recording to measure the amplitude and frequency of miniature excitatory postsynaptic currents (mEPSCs) in hippocampal neurons treated with PrP^Sc^. mEPSCs, which are recorded in the presence of TTX to block action potentials and picrotoxin to block GABA-evoked inhibitory currents, are a measure of spontaneous synaptic currents evoked by glutamate. We found that treatment of hippocampal neurons with purified PrP^Sc^, but not with mock-purified material from uninfected brains, caused a marked reduction in mEPSC frequency, and a less pronounced but statistically significant decrease in mEPSC amplitude (Fig 1A–1C). These effects were not observed in neurons derived from PrP knockout mice (*Prn-p*^0/0^), demonstrating that the functional as well as the morphological effects of PrP^Sc^ on synapses are entirely PrP^C^-dependent (Fig 1D–1F). Of note, PrP^Sc^ did not significantly affect the frequency or amplitude of miniature inhibitory postsynaptic currents (mIPSCs) (Fig 1G–1I). This result indicates that PrP^Sc^ is strikingly selective in its effects, targeting primarily excitatory and not inhibitory synapses.

For the electrophysiology experiments shown in Fig 1, we used a culture system that was slightly different from the one used to monitor dendritic spine retraction. In this system, neurons were grown at a higher density on the same substrate with supporting astrocytes, an arrangement which improves neuronal integrity during patch clamp recording. We confirmed that, when recordings were done on neurons cultured at low density over an astrocyte feeder layer, PrP^Sc^ also caused a reduction in mEPSC amplitude and frequency (S1 Fig).

### PrP^Sc^ causes loss of excitatory postsynaptic markers without affecting presynaptic markers

**Fig 2:**
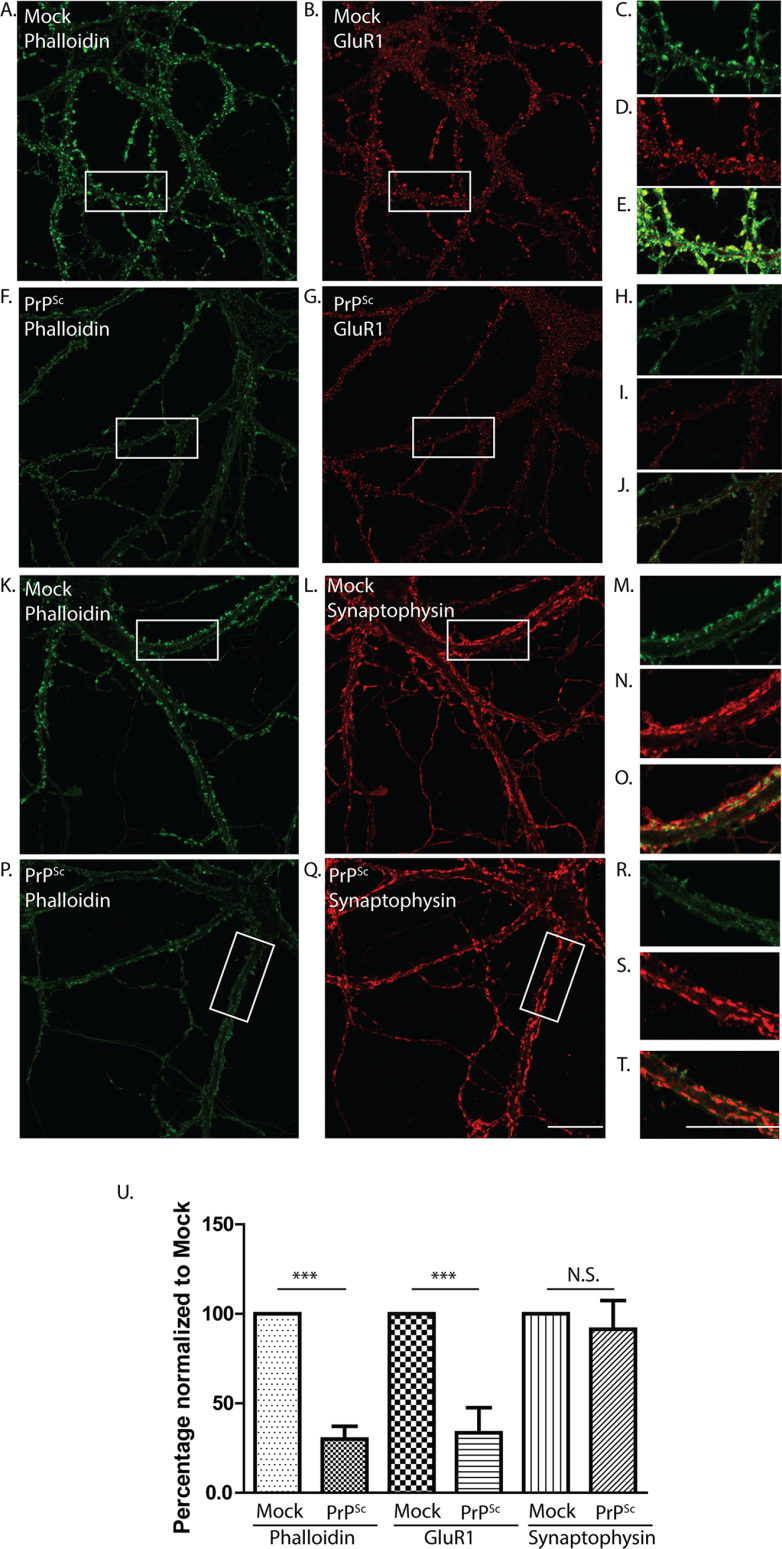
PrP^Sc^ causes loss of excitatory postsynaptic markers without affecting presynaptic markers. Hippocampal neurons were treated for 24 hrs with purified PrP^Sc^ (F, G, P, Q) or with mock-purified material (A, B, K, L). Cultures were then fixed and stained with fluorescent phalloidin (green) (A, F, K, P) along with antibodies to either GluR1 (excitatory postsynaptic marker) (B,G) or synaptophysin (presynaptic marker) (L, Q) (both red). The boxed regions in each pair of horizontal panels (A and B; F and G; K and L; P and Q) are shown at higher magnification in the pair of smaller panels to the right (C and D; H and I; M and N; R and S, respectively). Panels E, J, O, and T show merged green and red images for each pair of smaller panels. Quantitation of spine number of synaptic marker staining is shown in panel U, normalized to the values in mock-treated cultures. Pooled measurements were collected from 15–20 cells from 3 independent experiments. ***p<0.001 by Student’s t-test; N.S., not significantly different. Scale bar in panel Q = 20 μm (also applicable to panels A, B, F, G, K, L, and P); scale bar in panel T = 20 μm (also applicable to panels C-E, H-J, M-O, and R-S).

The reduction in mEPSC amplitude and frequency caused by PrP^Sc^ could be due to effects on either presynaptic processes (e.g. synaptic release) or postsynaptic characteristics (e.g., number and distribution of active zones). To distinguish presynaptic from postsynaptic effects, we immunostained neurons with antibodies to GluR1, an AMPA receptor subunit present in the postsynaptic density, and synaptophysin, a presynaptic marker. When neurons were treated with purified PrP^Sc^, but not mock-purified material, there was a dramatic loss of GluR1 staining, consistent with the retraction of dendritic spines revealed by fluorescent phalloidin staining (Fig 2A–2J and 2U). In contrast, there was no statistically significant loss of synaptophysin staining after PrP^Sc^ treatment (Fig 2K–2T and 2U). These data demonstrate that PrP^Sc^ exerts a highly selective effect on postsynaptic elements, with no detectable effect on presynaptic structures, even in the face of massive morphological changes in dendritic spines.

We also found that PrP^Sc^ treatment did not change the number of inhibitory synapses, as shown by staining for gephyrin (a postsynaptic anchoring component for glycine and GABA_A_ receptors) (S2 Fig). This result is consistent with the inhibitory effect of PrP^Sc^ on mEPSCs and not mIPSCs (Fig 1). We conclude that PrP^Sc^ primarily targets the postsynaptic elements of excitatory synapses in the neuronal culture systems we are using.

### PrP^Sc^ synaptotoxicity requires glutamate receptor activation, and is accompanied by calcium influx

**Fig 3:**
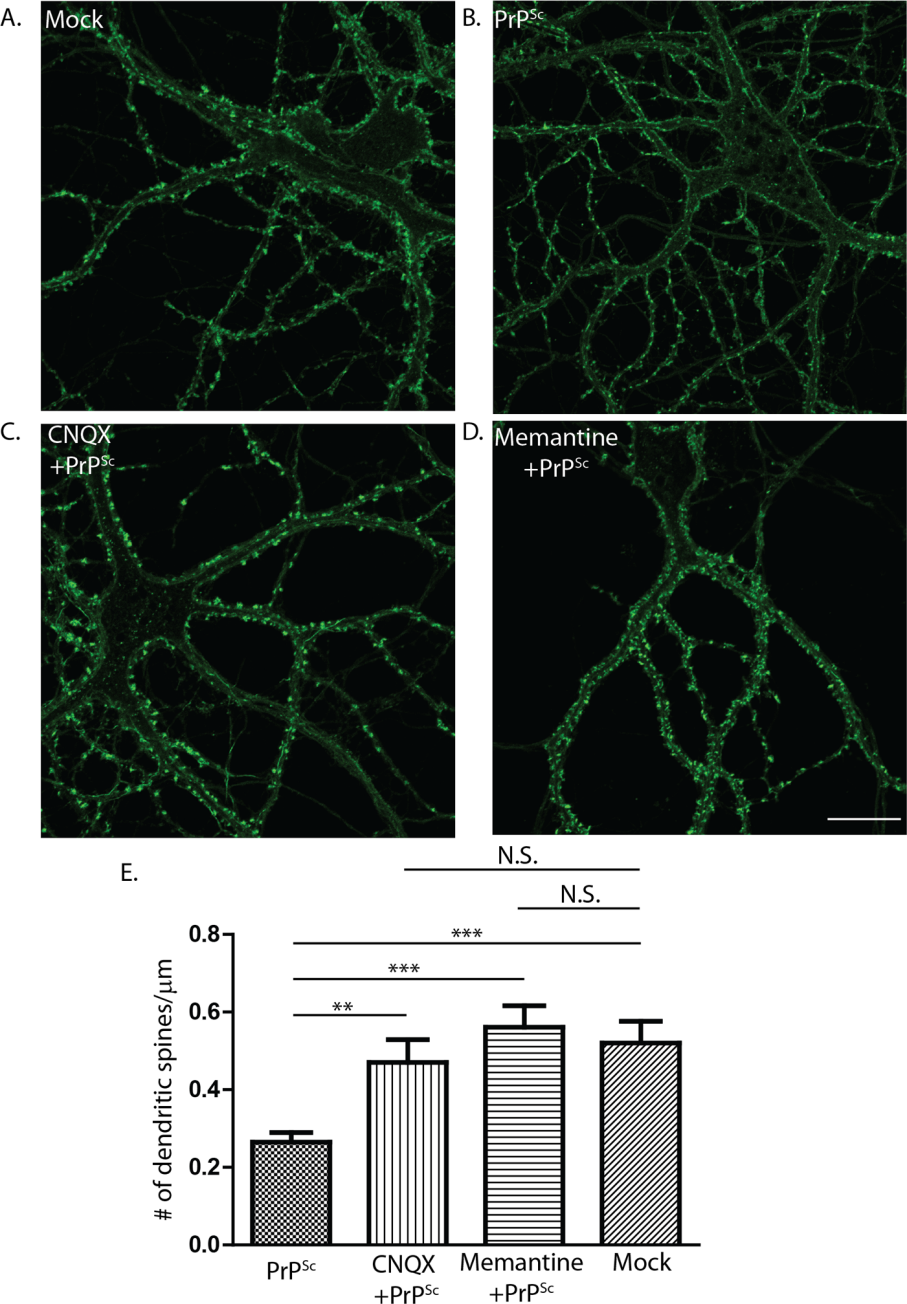
PrP^Sc^ synaptotoxicity requires activation of NMDA and AMPA receptors. Hippocampal neurons were treated for 24 hrs with purified PrP^Sc^ (B-D), or with mock-purified material from uninfected brains (A) in the absence of inhibitors (A, B) or in the presence of the AMPA receptor blocker CNQX (10 μM) (C) or the NMDA receptor blocker memantine (10 μM) (D). Neurons were then fixed and stained with fluorescent phalloidin to visualize dendritic spines. Pooled measurements of spine number were collected from 15–20 cells from 3 independent experiments (E). **p<0.01, ***p<0.001 by Student’s t-test; N.S., not significantly different. Scale bar in panel D = 20 μm (also applicable to panels A-C).

Glutamate receptor-dependent excitotoxicity contributes to the pathogenesis of many neurodegenerative diseases [26–30]. The selective effect of PrP^Sc^ on excitatory synapses suggested the possibility that ionotropic glutamate receptors might play a role in the degeneration of dendritic spines seen in this system. To test this possibility, we treated hippocampal cultures with purified PrP^Sc^, or with mock-purified material form uninfected brains, in the presence or absence of NMDA or AMPA receptor blockers (see Table 1 for a list of pharmacological inhibitors used in this study). We observed that the competitive AMPA receptor antagonist CNQX, as well as the uncompetitive NMDA channel blocker memantine, prevented PrP^Sc^-induced retraction of dendritic spines (Fig 3). Spine number in PrP^Sc^-exposed cultures in the presence of these drugs was not statistically different from cultures exposed to mock material from uninfected brains.

**Table 1:** Pharmacological inhibitors used in this study.

Inhibitor name	Molecular Target	Company*	Final concentration
	**Glutamate Receptors**		
CNQX	AMPA receptor	Tocris	10 μM
memantine	NMDA receptor	Sigma	10 μM
MPEP	mGluR5	Tocris	10 μM
	**Voltage-Gated Ca**^**2+**^ **Channels**		
SNX482	R type	Tocris	40 nm
w-Agatoxin TK	P/Q type	Tocris	500 nm
ML218	T type	Tocris	1 μM
w-Conotoxin GVIA	N type	Tocris	100 nm
Lomerizine	L type	Alomone Labs	5 μM
	**MAPK Signaling**		
SB239063	p38 MAPK	Tocris	10 μM
VX745	p38α MAPK	Tocris	100 nM
FR180204	ERK	Tocris	500 nM
SP600125	JNK	Tocris	10 μM
CAS1186648	MK	Millipore	500 nM
SB747651	MSK	Tocris	10 μM
ETP45835	MNK	Millipore	1 μM
	**Cytoskeleton**		
Taxol	Microtubules (stabilizer)	Tocris	10 μM
SiR-actin	F-actin (stabilizer)	Cytoskeleton Inc.	1 μM
	**Unfolded Protein Response**		
ISRIB	eIF2B	Tocris	20 nM
GSK2606414	PERK	APExBIO	500 nM

Stock solutions of drugs were prepared in DMSO, and were added to neuronal cultures such that the final DMSO concentration in the culture medium was <0.1%. Vehicle-treated cultures were incubated in medium containing an equivalent concentration of DMSO. Cultures were pre-treated with drug for 2 hrs, after which PrP^Sc^ or ADDLs were added and incubation was continued for a further 24 hrs in the continued presence of the drug.

*Company locations: Tocris (Minneapolis, MN); Sigma (St. Louis, MO); Alomone Labs (Jerusalem, Israel); Millipore (Burlington, MA); Cytoskeleton Inc. (Denver, CO); APExBIO (Houston, TX).

**Fig 4:**
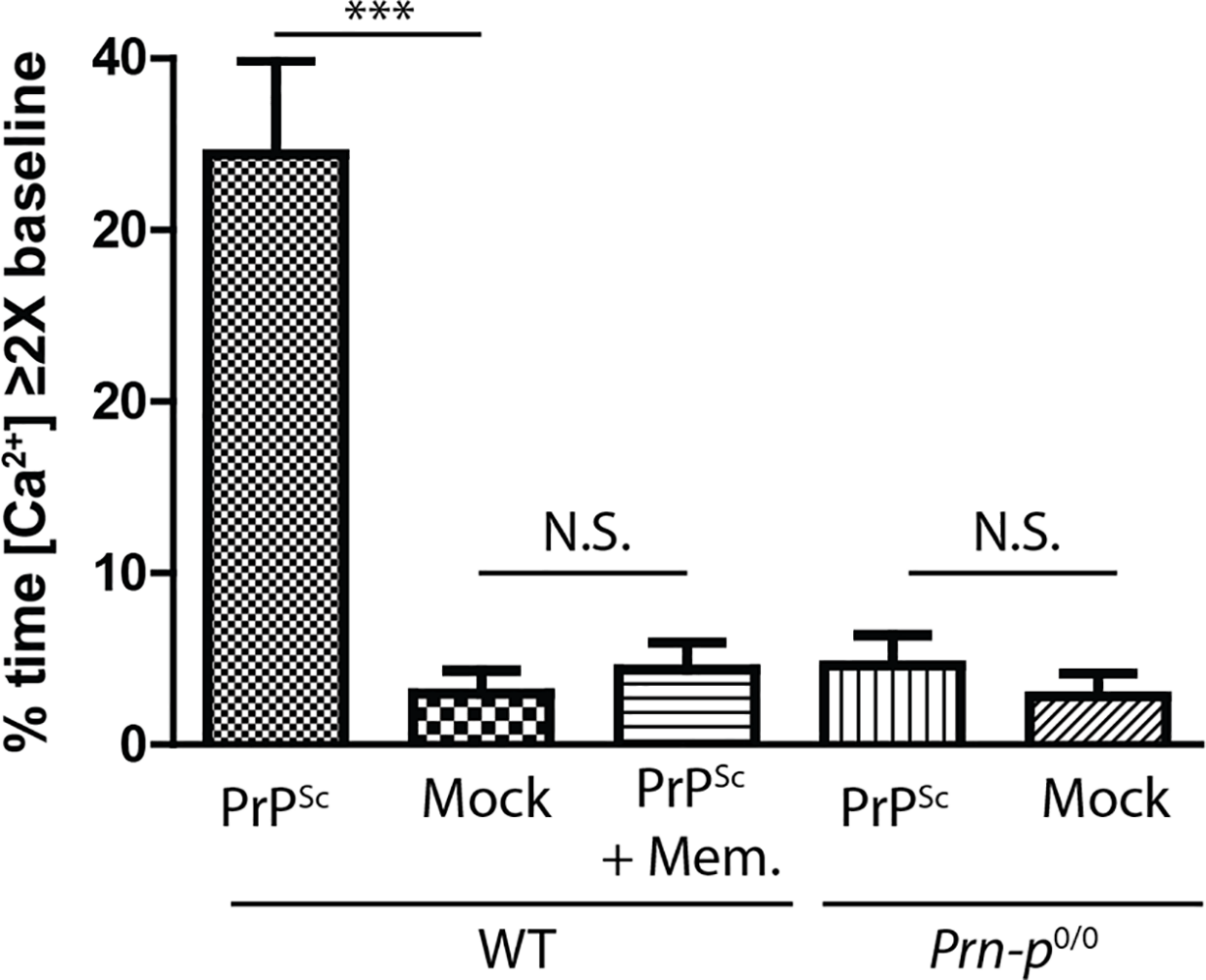
PrP^Sc^ induces NMDA receptor-dependent calcium influx in WT neurons but not in PrP knockout neurons. Hippocampal neurons from WT or *Prn-p*^0/0^ mice were treated with purified PrP^Sc^ or with mock-purified material in the presence or absence of the NMDA receptor blocker memantine (10 μM). Intracellular Ca^2+^ was monitored using Fluo-3. Each cell was imaged for 5 min before treatment to define the baseline calcium level, and then for 30 min after treatment. The proportion of the 30 min recording period during which the calcium signal was ≥ 2X the baseline level was plotted. N = 15–20 cells from 3 independent experiments. ***p<0.001 by Student’s t-test; N.S., not significantly different. Mem. = memantine.

Glutamate receptor-mediated excitotoxicity is often accompanied by an influx of Ca^2+^ ions via NMDA receptors. To test whether this mechanism was operative during PrP^Sc^ synaptotoxicity, we used the calcium-sensitive dye Fluo-3 to image intracellular calcium levels in neurons treated with PrP^Sc^. We found that purified PrP^Sc^, but not mock-purified material, caused a significant increase in intracellular calcium (Fig 4). The effect of PrP^Sc^ on Ca^2+^ levels was absent in neurons derived from *Prn-p*^0/0^ mice lacking PrP^C^, and was completely blocked by memantine. Since voltage-gated calcium channels (VGCCs) are also major mediators of calcium influx into dendritic spines, we tested the effect of inhibitors of the major classes of VGCCs (R, T, N, P/Q, and L type). None of these inhibitors had a statistically significant effect on PrP^Sc^-induced reduction in dendritic spine numbers, with the exception of lomerizine (an L-type VGCC inhibitor), which had a partial protective effect (S3 Fig).

We conclude from these data that activation of NMDA and AMPA receptors plays an essential role in dendritic spine retraction induced by PrP^Sc^, and that the effect of PrP^Sc^ is accompanied by Ca^2+^ influx, primarily via NMDA receptors.

### Inhibition of p38 MAPK, but not ERK nor JNK, prevents PrP^Sc^ synaptotoxicity

**Fig 5:**
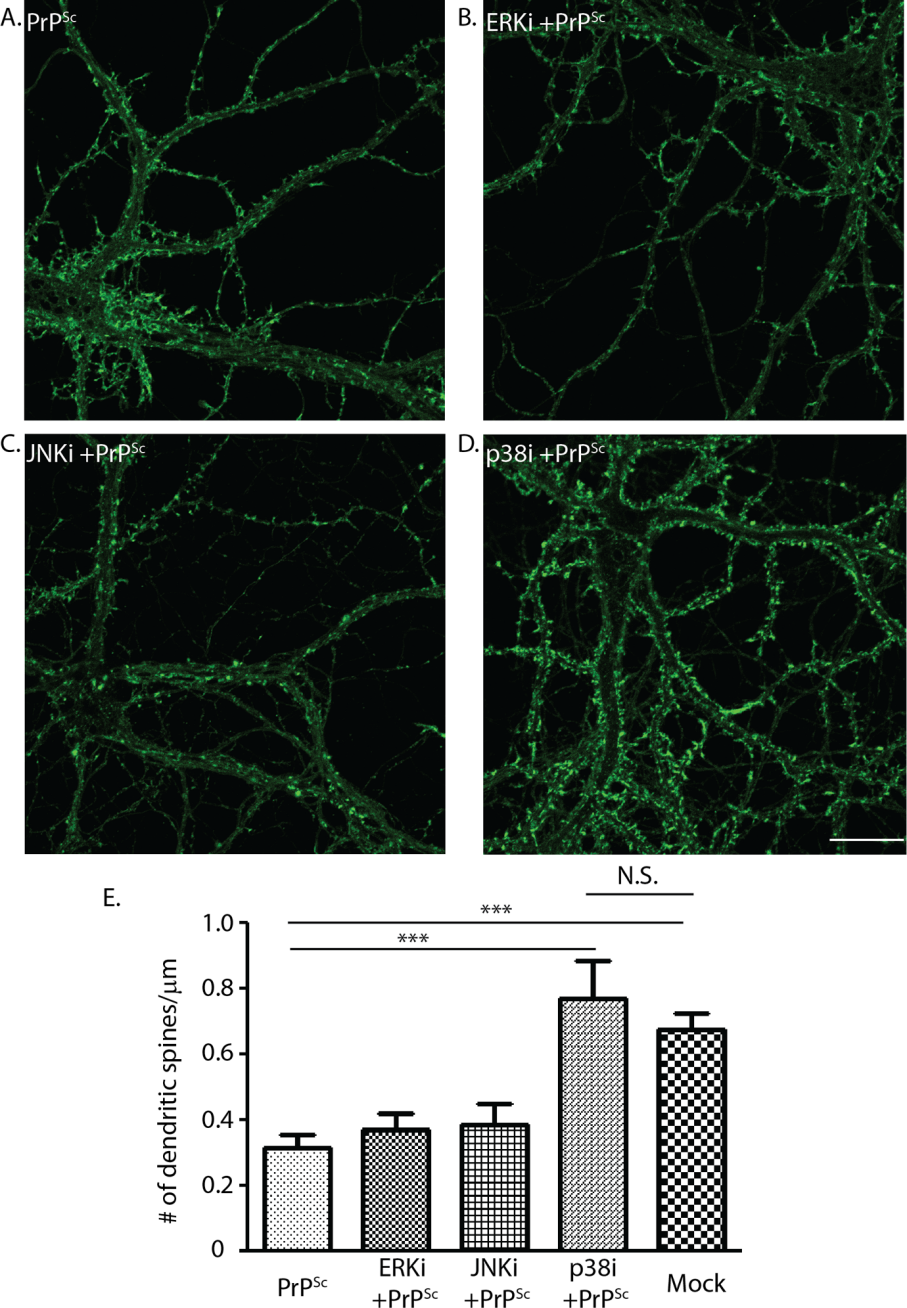
A p38 MAPK inhibitor, but not ERK or JNK inhibitors, prevents PrP^Sc^-induced retraction of dendritic spines. Hippocampal neurons were treated for 24 hrs with purified PrP^Sc^ in the absence of inhibitors (A), or in the presence of an ERK inhibitor (FR180204, 500 nM) (B), a JNK inhibitor (SP600125, 10 μM) (C), or a p38 MAPK inhibitor (SB239063, 10 μM) (D). Pooled measurements of spine number were collected from 15–20 cells from 3 independent experiments (E). ***p<0.001 by Student’s t-test; N.S., not significantly different. The bar labeled Mock represents cultures treated with mock-purified material in the absence of inhibitors. Scale bar in panel D = 20 μm (also applicable to panels A-C).

**Fig 6:**
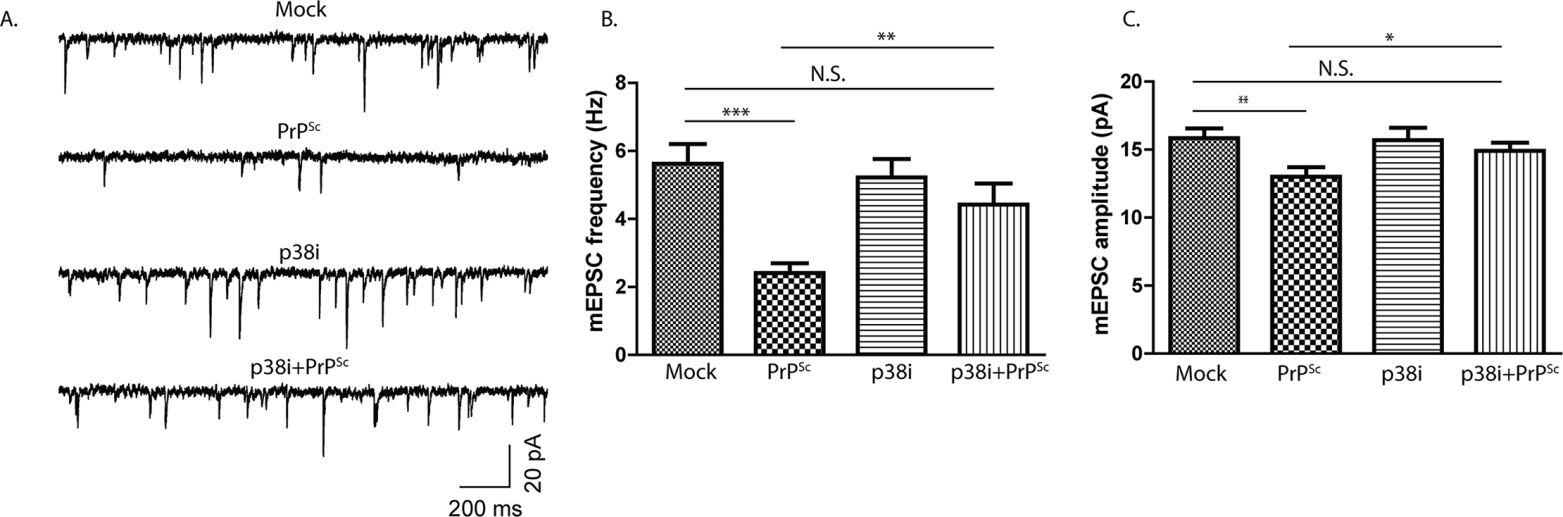
A p38 MAPK inhibitor prevents PrP^Sc^-induced reduction in spontaneous synaptic neurotransmitter release. Hippocampal neurons were treated for 24 hrs with either mock-purified material, purified PrP^Sc^, a p38 MAPK inhibitor (SB239063, 10 μM) alone, or the p38 MAPK inhibitor in the presence of purified PrP^Sc^. Whole-cell patch clamping was used to record mEPSCs (A), the frequencies and amplitudes of which were quantitated (B,C). N = 15 cells from 3 independent experiments. ***p<0.001, **p<0.01, *p<0.05 by Student’s t-test; N.S., not significantly different.

Mitogen-activated protein kinases (MAPKs) are important signal transducers downstream of many kinds of intracellular and extracellular stimuli [31], including stressful stimuli like excitotoxicity [32]. In mammals, the MAPKs are grouped into three main families, ERKs (extracellular-signal-regulated kinases), JNKs (Jun amino-terminal kinases), and p38/SAPKs (stress-activated protein kinases) [31]. We tested whether any of these MAPK families are involved in PrP^Sc^ synaptotoxicity. We observed that a p38 MAPK inhibitor (SB239063), which targets all four isoforms (α, β, γ, δ), effectively prevented spine retraction caused by PrP^Sc^, while pan-isoform inhibitors of ERK and JNK were without effect (Fig 5). The inhibitors alone had no effect on spine number. In parallel with its ability to block the effects of PrP^Sc^ on spine morphology, we found that the p38 MAPK inhibitor prevented the PrP^Sc^-induced reduction in mEPSC frequency and amplitude (Fig 6). Taken together, these data indicate that p38 MAPK plays an essential role in mediating the toxic effects of PrP^Sc^ on synaptic structure and function in this system.

Mammalian p38 MAPK has four isoforms (α, β, γ, and δ) [33]. p38γ is most highly expressed in skeletal muscle, and p38δ in testis, pancreas, kidney and small intestine [34]. Thus, p38α and p38β are the isoforms most likely to be involved in neuronal signaling. The α and β isoforms are 75% identical, and both are inhibited by the compound SB239063 used in Figs 5 and 6. To determine which of the two p38 isoforms is involved in PrP^Sc^ synaptotoxicity, we utilized the p38α -specific inhibitor VX745. We found that VX745 completely blocked the effects PrP^Sc^ on dendritic spine number and mEPSC properties, suggesting that p38α is one of the isoforms involved (S4 Fig). The absence of a p38β-specific inhibitor precluded specific testing of this isoform.

### PrP^Sc^-induced retraction of dendritic spines is reversible by application of a p38 MAPK inhibitor

**Fig 7:**
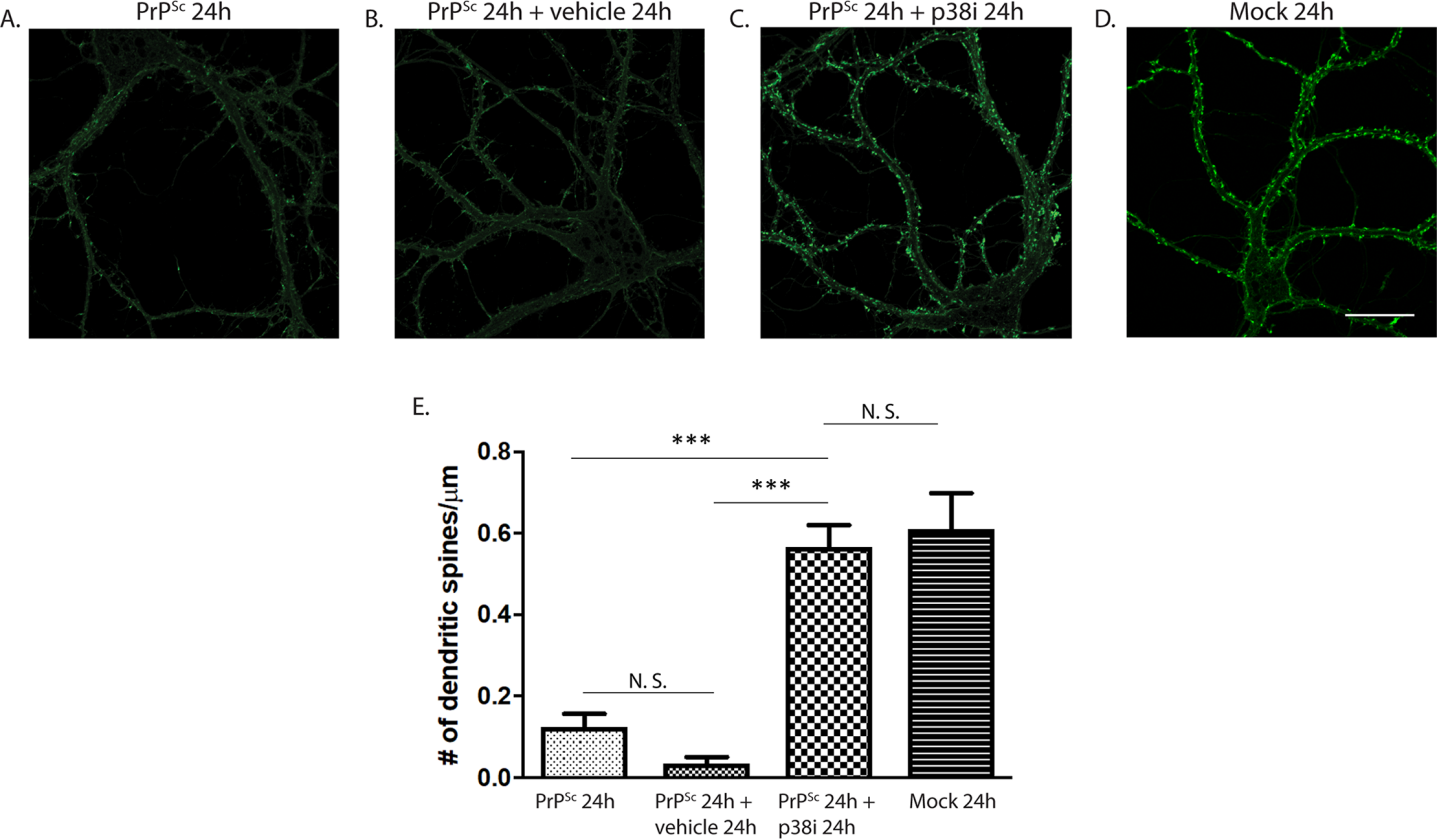
PrP^Sc^ synaptotoxicity is reversible by a p38 MAPK inhibitor. Hippocampal neurons were treated for 24 hrs with purified PrP^Sc^ (A-C). One set of cultures (A) was then fixed and stained with fluorescent phalloidin; a second and third set were treated with PrP^Sc^ for an additional 24 hrs in the presence of vehicle (B) or a p38 MAPK inhibitor (SB239063, 10 μM) (C). A fourth set of cultures (D) was treated for 24 hrs with mock-purified material. All cultures were then fixed and stained with phalloidin. Pooled measurements of dendritic spine number were collected from 15–20 cells from 3 independent experiments (E). ***p<0.001 by Student’s t-test; N.S., not significantly different. Scale bar in panel D = 20 μm (also applicable to panels A-C).

Because abnormalities in dendritic spines and synaptic transmission are early effects of PrP^Sc^, which occur well before large-scale changes in neuronal morphology or loss of neuronal viability, we wondered whether the effects of PrP^Sc^ might be reversible by treatment with a p38 MAPK inhibitor. To test this possibility, we treated neurons with PrP^Sc^ for 24 hrs, at which point most of the dendritic spines were retracted (Fig 7A). We then exposed the neurons to PrP^Sc^ for additional 24 hrs in the presence of a p38 MAPK inhibitor (SB239063) or vehicle control, after which cultures were fixed and assessed for dendritic spine morphology with fluorescent phalloidin. Amazingly, we found that the p38 MAPK inhibitor was able to reverse the dendritic spine retraction that had accrued during the first 24 hrs of PrP^Sc^ treatment (Fig 7C), compared to the cultures treated with vehicle (Fig 7B). Quantitation of spine number under the three conditions is shown in Fig 7E. These data indicate that the extensive dendritic spine abnormalities induced by PrP^Sc^ are reversible by p38 MAPK inhibition, at least within a 48 hr time window.

### A dominant-negative mutant of p38 MAPK prevents PrP^Sc^ synaptotoxicity

**Fig 8:**
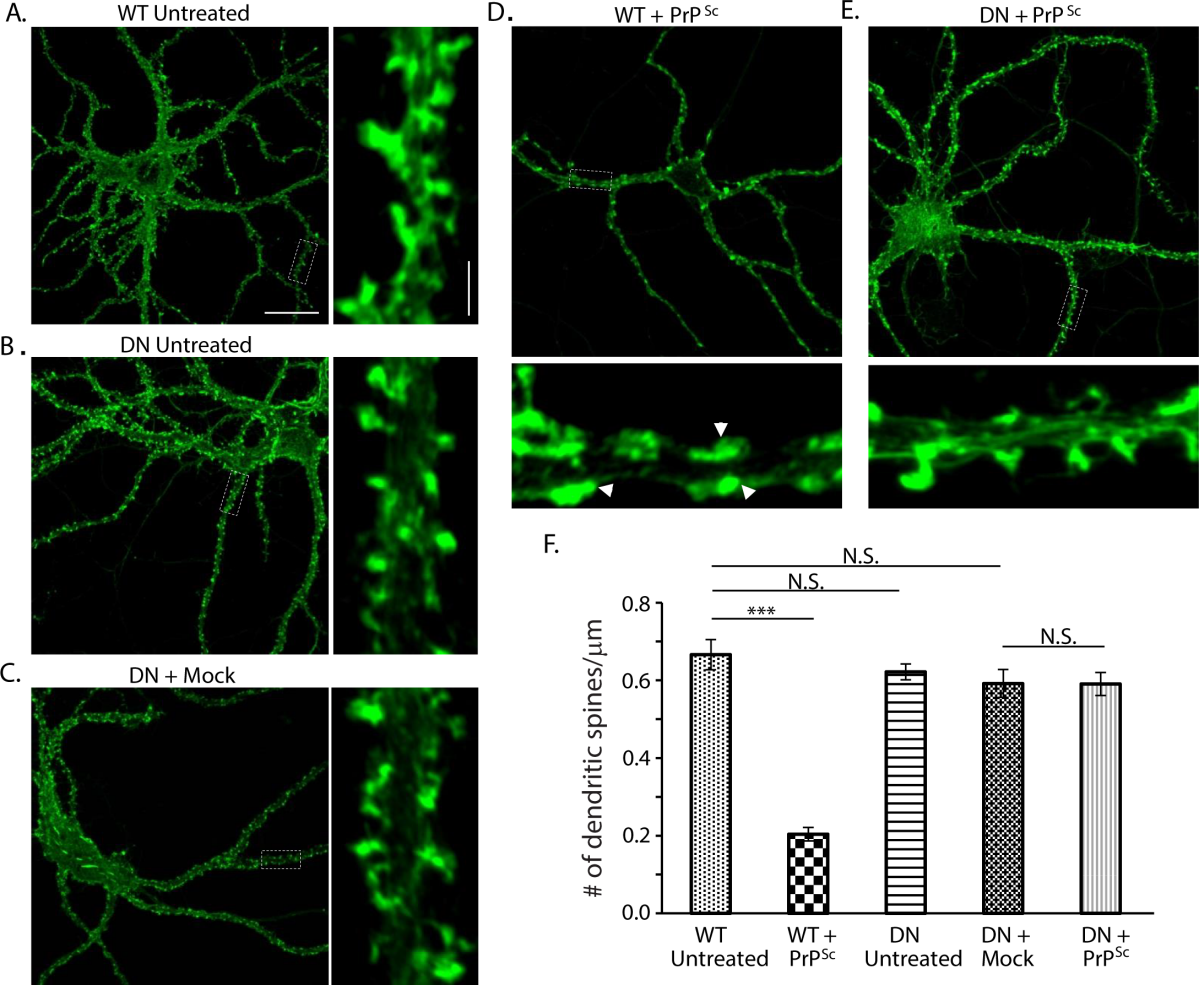
A dominant-negative mutant of p38 MAPK prevents PrP^Sc^ synaptotoxicity. Hippocampal neurons from WT mice (A, D) or p38AF dominant-negative (DN) mice (B, C and E) mice were untreated (A, B), or were exposed to purified PrP^Sc^ (D, E) or mock purified material (C) for 24 hrs. Neurons were then fixed and stained with fluorescent phalloidin to visualize dendritic spines. The boxed regions in each panel are shown at higher magnification in the smaller panels to the right (A-C) and bottom (D, E). Arrowheads in the higher magnification panels in panel D show the positions of collapsed spines. Pooled measurements of spine number were collected from 15–20 cells from 4 animals (F). ***p<0.001 by Student’s t-test; N.S., not significantly different. Scale bars in panel A = 20 μm (main image) and 2 μm (higher magnification image) (also applicable to panels B-E).

To confirm the results obtained with pharmacological inhibition of p38 MAPK, we employed a genetic method to suppress signaling through this pathway, which makes use of a dominant-negative form of p38α MAPK (T180A/Y182F, referred to as p38AF). This double-mutation in the activation loop of the kinase prevents phosphorylation by upstream kinases, and has a dominant-negative effect on the activity of co-expressed wild-type p38, thereby significantly attenuating signaling [35]. We prepared hippocampal neurons from mice that were heterozygous for the p38AF allele. This method of reducing p38 signaling avoids the embryonic lethal phenotype that results from complete germline inactivation of the p38 MAPK gene [36]. We found that neurons prepared from p38AF mice were morphologically comparable to WT neurons, but were almost completely resistant to the dendritic spine retraction effect of PrP^Sc^ (Fig 8). This experiment, which depends on constitutive down-regulation of p38 signaling, complements the previous experiments, which involved acute, pharmacological inhibition of p38 MAPK activity.

### PrP^Sc^ causes increased phosphorylation of p38 MAPK in dendritic spines

**Fig 9:**
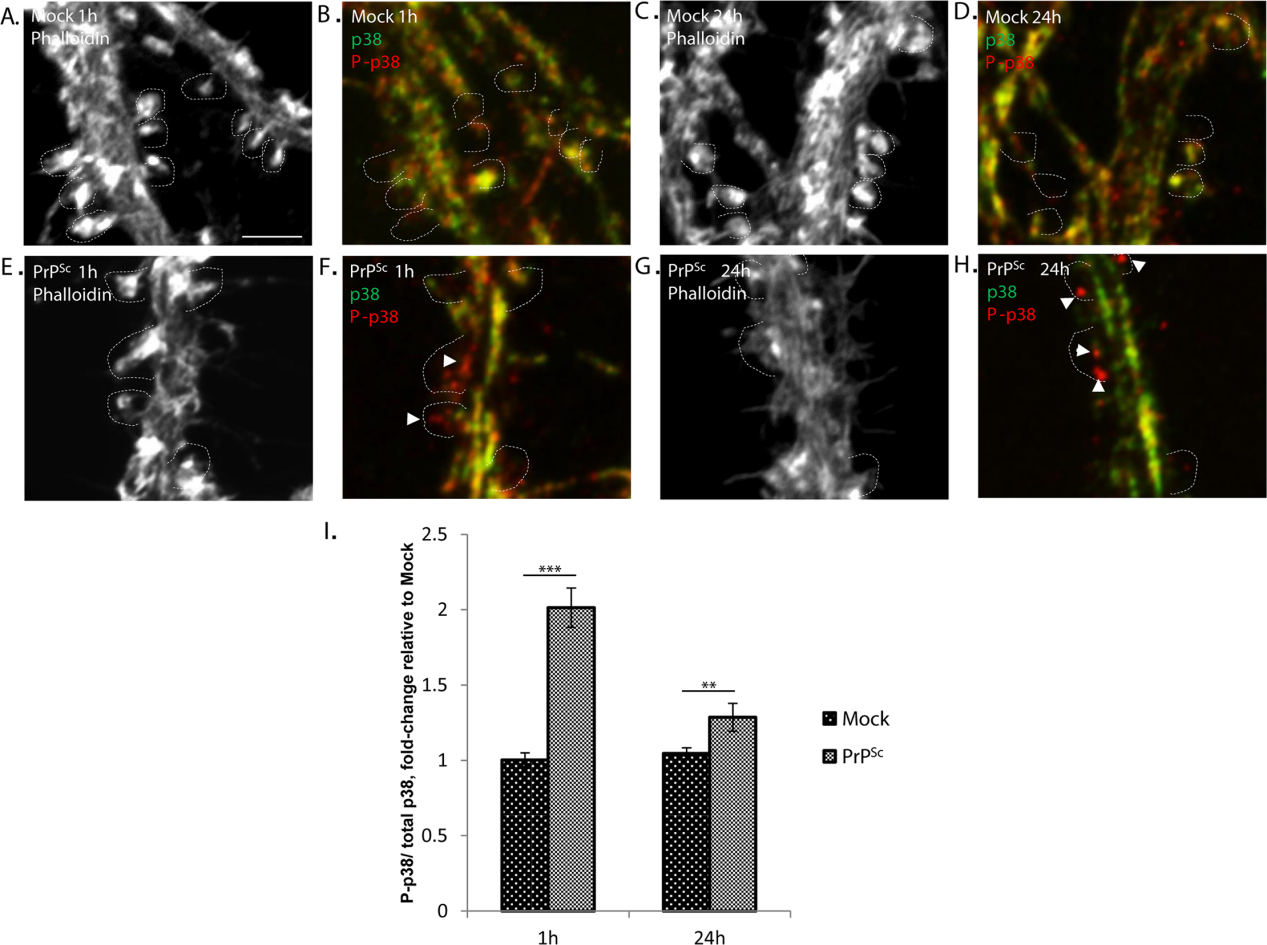
PrP^Sc^ causes increased phosphorylation of p38 MAPK in dendritic spines. Hippocampal neurons were treated for 1 hour (A, B, E, F) or 24 hours (C, D, G, H) with either mock-purified material (A-D) or purified PrP^Sc^ (E-H). Cultures were then fixed and stained with fluorescent phalloidin, along with antibodies to total p38 MAPK and phospho-p38 MAPK (P-Thr180/P-Tyr182). Panels A, E, C, and G show images of phalloidin staining. Panels B, F, D, and H show merged images of total p38 (green pseudo-color) and phospho-p38 (red pseudo-color) staining. Dotted lines outline the positions of intact spines (A-F) or collapsed spines (G, H), based on phalloidin staining. Arrowheads in panels F and H indicate regions (red) of enhanced phospho-p38/total p38 staining. Pooled measurements of the ratio of phospho-p38 to total p38 staining were collected from 20–30 dendritic spine regions from 3–4 independent experiments (I). ***p<0.001, **p<0.01 by Student’s t-test. Scale bar in panel A = 2 μm (also applicable to panels B-H).

To provide biochemical evidence for activation of the p38 MAPK pathway in response to PrP^Sc^, we utilized an immunocytochemical approach in order to detect localized changes in p38 phosphorylation. We doubly stained PrP^Sc^-treated cultures with antibodies to phospho-p38 and total p38, and then imaged the ratio of the two signals in the region of dendritic spines, as marked by phalloidin staining. We found that the amount of phosphorylated p38 in dendritic spines was increased after 1 hr of PrP^Sc^ treatment, and remained elevated after 24 hrs in the region of collapsed spines (Fig 9).

### MAPK-activated protein kinases (MKs) are downstream targets of p38 MAPK in the PrP^Sc^ synaptotoxic pathway

**Fig 10:**
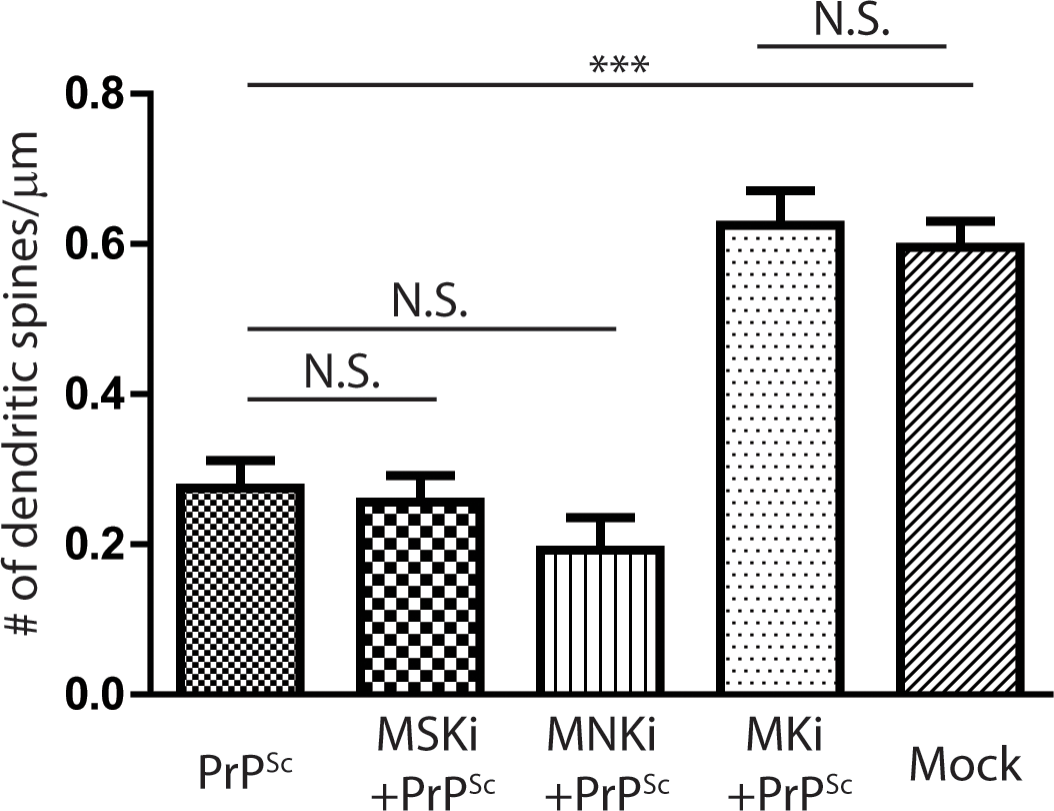
MAPK-activated protein kinases (MKs) are downstream targets of p38 MAPK in the PrP^Sc^ synaptotoxic pathway. Hippocampal neurons were treated for 24 hrs with purified PrP^Sc^ in the absence of inhibitors, or in the presence of inhibitors of MSK1/2 (SB747651, 10 μM), MNK1/2 (ETP45835, 1 μM), or MK2/3/5 (CAS1186648, 500 nM). Pooled measurements of spine number were collected from 15–20 cells from 3 independent experiments. ***p<0.001 by Student’s t-test; N.S., not significantly different. The bar labeled Mock represents cultures treated with mock-purified material in the absence of inhibitors.

Known downstream targets of p38 MAPK include MAPK-activated protein kinase (MAPKAP or MK), MSK and MNK [reviewed in 37]. To determine which of these kinases is downstream of p38 MAPK in the PrP^Sc^ synaptotoxic pathway, neurons were treated with PrP^Sc^ in the presence or absence of specific inhibitors. We found that a pan MK inhibitor (CAS1186648), which targets all three isoforms (2, 3, and 5), effectively prevented PrP^Sc^-induced retraction of dendritic spines, while pan-isoform inhibitors of MSK and MNK had no significant effect (Fig 10). None of the three inhibitors alone had a significant effect on spine number. We conclude from these results that MK2, 3, or 5 are potential targets of p38 MAPK in the pathway leading to dendrite retraction caused by PrP^Sc^. Of these, MK2 and MK3 are the most likely isoforms to be involved, since MK5 is expressed primarily in the heart, while MK2 and MK3 are ubiquitously expressed [38, 39]. MK2/3 are both substrates for the α isoform of p38 MAPK.

### p38 MAPK and MK inhibitors do not affect PrP^Sc^ propagation in ScN2a cells

One possible mechanism by which inhibitors of MAPK pathways could prevent PrP^Sc^-induced synaptotoxicity would be by inhibiting conversion of endogenous neuronal PrP^C^ to PrP^Sc^, on the assumption that newly formed PrP^Sc^ is the primary trigger for synaptic degeneration. To address this possibility, we tested whether these inhibitors affected PrP^Sc^ formation in scrapie-infected N2a (ScN2a) cells. We found that neither the p38 MAPK inhibitor SB239063, nor the MK2/3/5 inhibitor CAS1186648, had a significant effect on the levels of protease-resistant PrP^Sc^ in ScN2a cells after 7 days of treatment (S5 Fig). As a positive control, the known anti-prion agent Congo red [40, 41], dramatically reduced PrP^Sc^ levels under the same conditions. None of the compounds had a significant cytotoxic effect, as indicated by measurement of the total amount of cellular protein. These results support the notion that the p38 MAPK and MK inhibitors block PrP^Sc^ synaptotoxicity by interfering with signaling pathways linked to synaptic integrity, rather than by reducing the amount of the toxic agent (PrP^Sc^).

### Actin stabilization counteracts the synaptotoxic effects of PrP^Sc^

**Fig 11:**
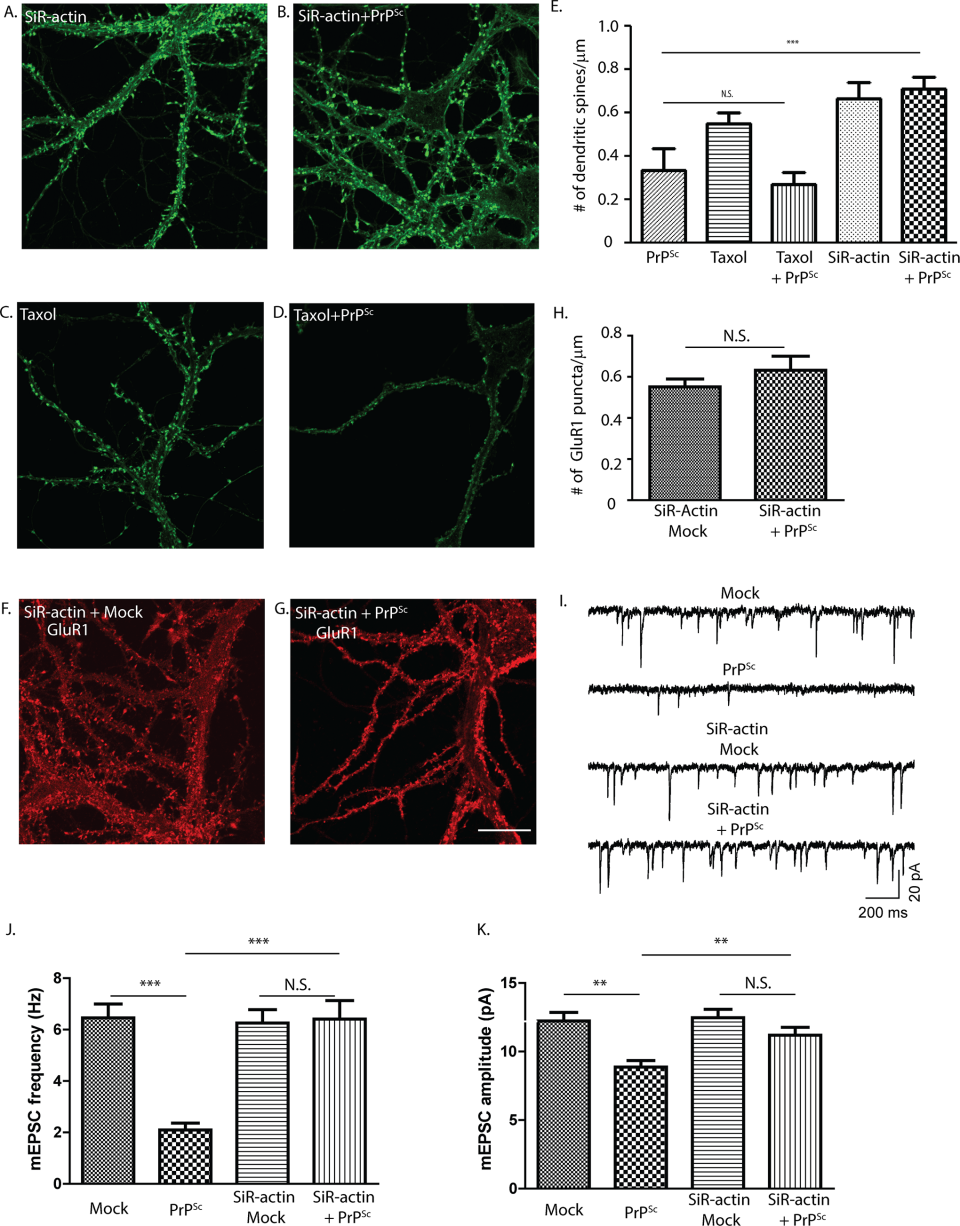
Actin destabilization is required for PrP^Sc^ synaptotoxicity. Hippocampal neurons were treated for 24 hrs with SiR-actin (1 μM) (A) or taxol (10 μM) (C) alone, or in combination with PrP^Sc^ (B, D, respectively). Cultures treated with SiR-actin (A, B) were fixed and imaged directly, and those treated with taxol (C, D) were fixed and stained with fluorescent phalloidin prior to imaging. Pooled measurements of dendritic spines were collected from 15–20 cells from 3 independent experiments (E). In a separate set of experiments, neurons were treated for 24 hrs with SiR-actin in the presence of mock-purified material (F) or purified PrP^Sc^ (G). Cultures were then fixed and stained with an antibody to GluR1. Pooled measurements of GluR1 staining were collected from 15–20 cells from 3 independent experiments. (For comparison, GluR1 staining of neurons in the absence of SiR-actin is shown in Fig 2A–2J and 2U). A third set of cultures was treated for 24 hrs with mock-purified material or purified PrP^Sc^ in the absence or presence of SiR-actin, and was then analyzed by patch clamping to measure mEPSC frequency and amplitude (I-K). N = 20 cells from 4 independent experiments. In E, H J, K: ***p<0.001 and **p<0.01 by Student’s t-test; N.S., not significantly different. Scale bar in panel G = 20 μm (also applicable to panels A-D and F).

Actin is abundant in dendritic spines and has been shown to regulate spine morphology [42]. In addition, there is evidence that most signaling pathways linking synaptic activity to spine morphology influence local actin dynamics [43, 44]. To address the role of actin in PrP^Sc^-induced alterations in dendritic spine morphology, we used SiR-actin, a fluorogenic, cell-permeable peptide derived from jasplakinolide that both stabilizes and labels F-actin [45]. Neurons were treated with SiR-actin alone or in combination with PrP^Sc^, after which spines were visualized using SiR-actin fluorescence. For comparison, we exposed cultures to taxol, a microtubule-stabilizing agent, with or without PrP^Sc^, after which spines were visualized using fluorescent phalloidin staining. We found that SiR-actin, but not taxol, prevented PrP^Sc^-induced spine retraction (Fig 11A–11E).

In a second experiment, we treated cultures with SiR-actin in the presence of PrP^Sc^ or mock-purified material, and then stained them with an antibody to the AMPA receptor subunit GluR1. We observed that SiR-actin prevented loss of GluR1 staining in response to PrP^Sc^ (Fig 11F–11H), parallel to the effect of this compound on spine retraction monitored using SiR-actin fluorescence (Fig 11A, 11B and 11E).

Consistent with these microscopic imaging results, electrophysiological recordings showed that SiR-actin prevented the decreases in mEPSC amplitude and frequency caused by PrP^Sc^ treatment (Fig 11I–11K).

Taken together, these data demonstrate that actin dynamics play an important role in the morphological changes in dendritic spines induced by PrP^Sc^, and that stabilizing actin filaments can prevent these changes.

### The unfolded protein response (UPR) does not play a prominent role in PrP^Sc^ synaptotoxicity

Activation of the UPR has been suggested to play a role in the pathogenesis of prion diseases, and blocking this pathway has been shown to produce a therapeutic benefit [46–48]. To address whether the UPR plays a role in PrP^Sc^-induced synaptotoxicity in our system, we treated cultured neurons with an inhibitor of PERK kinase (GSK2606414) or an activator of eIF2B (ISRIB [49, 50]), both of which reverse the eIF2α-mediated translational repression that occurs during the UPR. Neither compound had a significant effect on spine retraction induced by PrP^Sc^ (S6 Fig), suggesting that the UPR, in particular the translational repression arm mediated by PERK and eIF2α, does not play a role in PrP^Sc^ synaptotoxicity in this system.

### PrP^Sc^ and Aβ oligomers activate different molecular pathways

It has been proposed that PrP^C^ is a cell-surface receptor for Aβ oligomers, which mediates some of the neurotoxic effects of these assemblies, including loss of dendritic spines [51–55]. There is evidence that binding of Aβ oligomers to PrP^C^ triggers a signaling pathway involving mGluR5 and Fyn kinase, and that preventing activation of these molecules using specific inhibitors prevents Aβ neurotoxicity and ameliorates neurological symptoms in mice [56–59]. Since the synaptotoxic effects of both PrP^Sc^ and Aβ are dependent on the expression of PrP^C^ as a cell surface receptor in target neurons, we asked whether both toxic aggregates activated the same cellular pathways downstream of PrP^C^.

**Fig 12:**
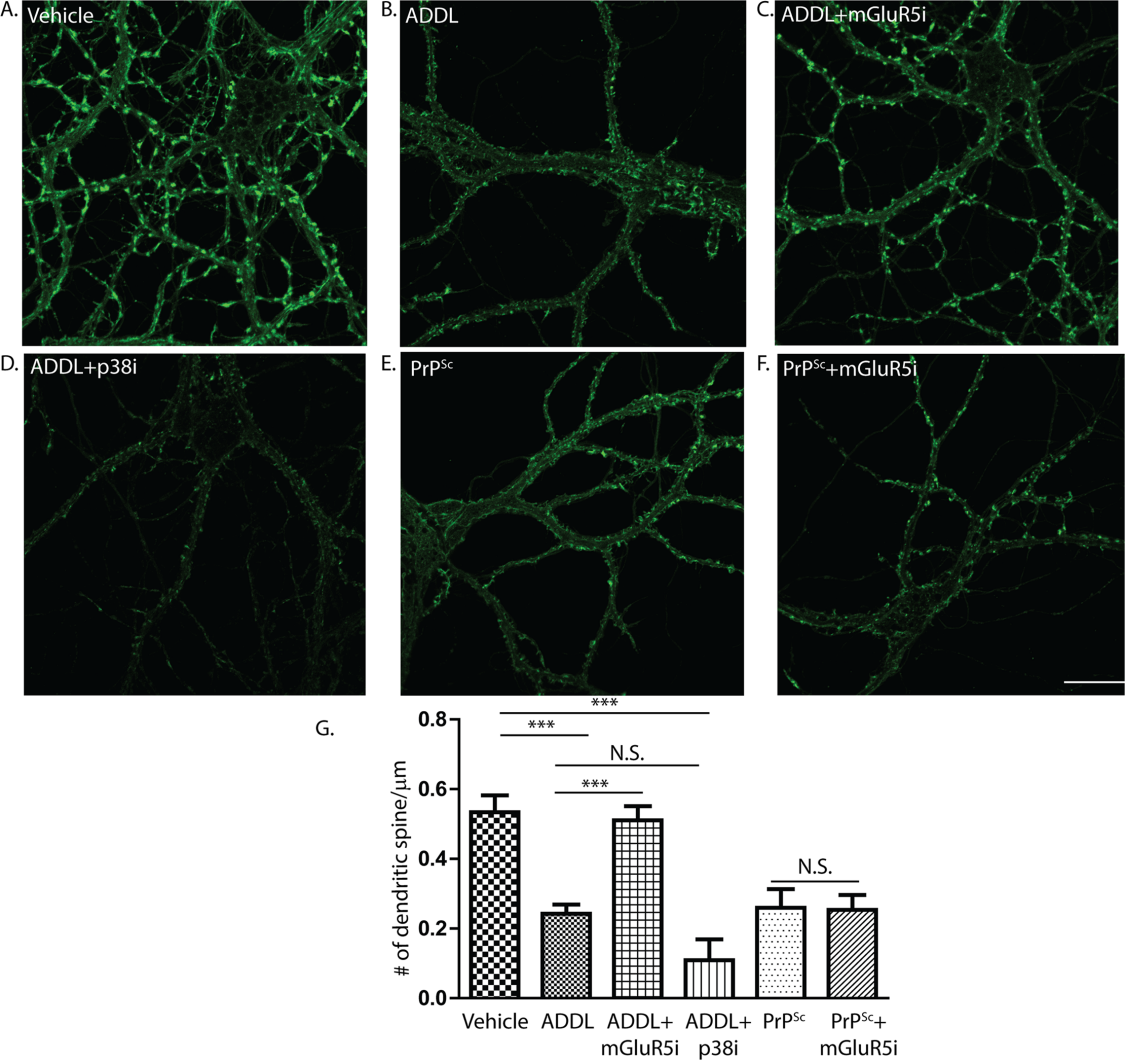
PrP^Sc^ and Aβ oligomers activate different signaling pathways. Hippocampal neurons were treated for 24 hrs with vehicle (A), with ADDLs (500 nM) in the absence of inhibitors (B), with ADDLs in the presence of an mGluR5 inhibitor (MPEP, 10 μM) (C), or with ADDLS in the presence of a p38 MAPK inhibitor (SB239063, 10 μM) (D). Another set of cultures was treated for 24 hrs with purified PrP^Sc^ in the absence of inhibitor (E) or in the presence of the mGluR5 inhibitor (F). Neurons were then fixed and stained with fluorescent phalloidin. Pooled measurements of dendritic spine number were collected from 15–20 cells from 3 independent experiments (G). ***p<0.001 by Student’s t-test; N.S., not significantly different. Scale bar in panel F = 20 μm (also applicable to panels A-E).

To investigate this question, we treated neurons with either Aβ or PrP^Sc^ in the presence or absence of the mGluR5 inhibitor, MPEP, or the p38 MAPK inhibitor, SB239063. Consistent with previously published studies [57], Aβ oligomers alone caused a significant reduction in dendritic spine number (Fig 12A and 12B), an effect that was dependent on expression of PrP^C^ (S7 Fig). MPEP completely blocked this effect (Fig 12C), also in agreement with published results [56]. In contrast, MPEP had no influence on PrP^Sc^-induced retraction of dendritic spines (Fig 12E and 12F). Moreover, the p38 MAPK inhibitor, which completely blocked PrP^Sc^ synaptotoxicity (Fig 5), had no significant effect on Aβ oligomer-induced dendritic spine loss (Fig 12D). Taken together, these data suggest that Aβ oligomers and PrP^Sc^ trigger different neurotoxic signaling pathways downstream of a common cell-surface receptor, PrP^C^.

## Discussion

**Fig 13:**
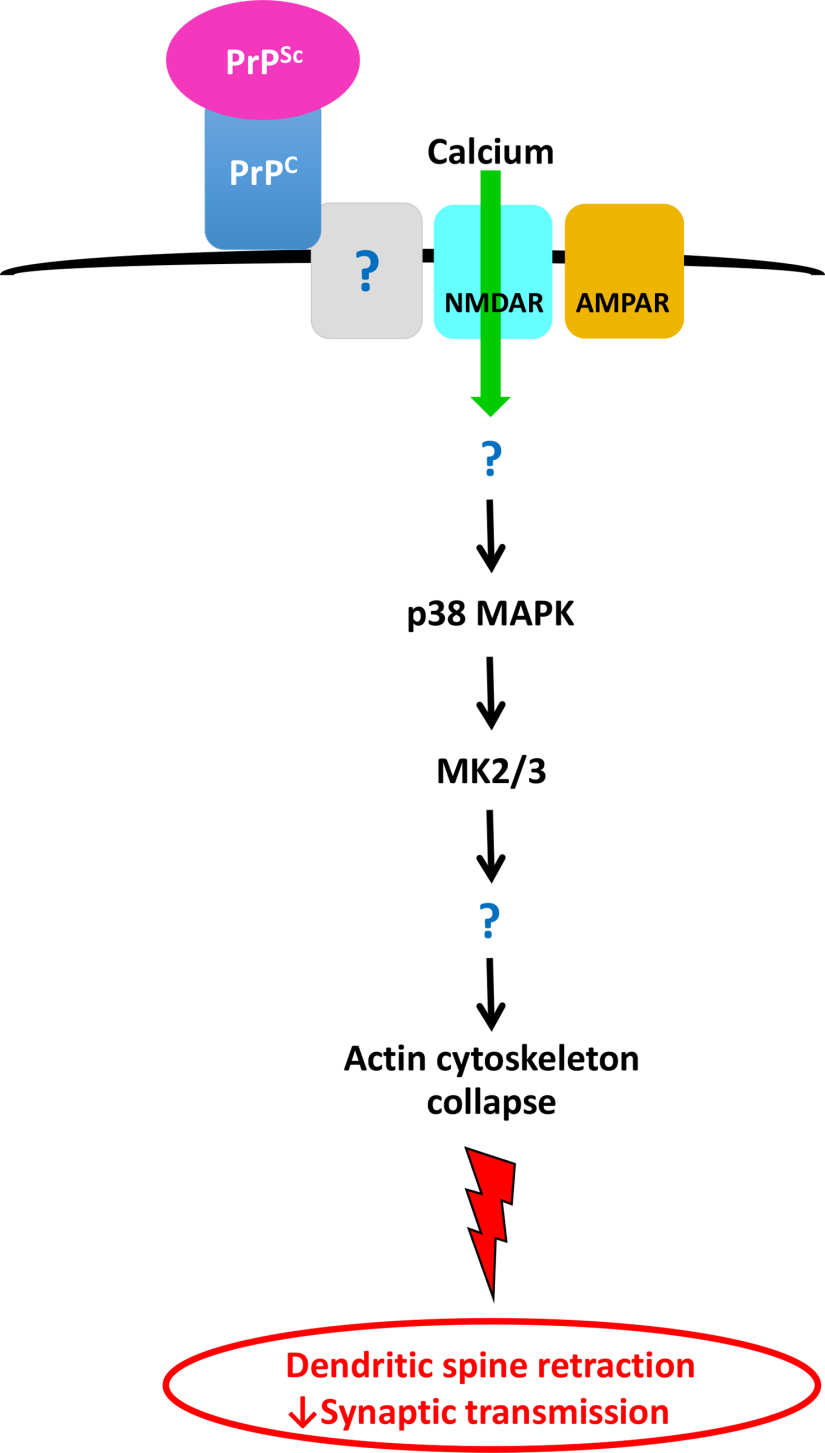
Model for a prion synaptotoxic pathway. The pathway is initiated by binding of PrP^Sc^ to endogenous, cell-surface PrP^C^. This binding event itself, or the subsequent conversion of PrP^C^ to PrP^Sc^, results in activation of NMDA and AMPA receptors with influx of calcium ions. Calcium influx leads to subsequent activation of p38 MAPK and MK2/3, collapse of the dendritic spine cytoskeleton, spine retraction, and decreases in synaptic transmission. Question marks indicate unknown components of the pathway.

Although the molecular templating process underlying the infectivity of prions is now well understood, the mechanisms by which prions cause neurodegeneration, in particular, damage to synapses, remain poorly understood. In a previously published study [23], we established a neuronal culture system that recapitulates one of the earliest events in prion synaptotoxicity, PrP^Sc^-induced retraction of dendritic spines. In the present work, we have exploited the simplicity of this system to dissect the cellular pathways underlying the toxic effects of PrP^Sc^ on synapses. Our results uncover a multi-step signaling cascade that begins with binding of PrP^Sc^ to PrP^C^ on the cell surface, and is followed by activation of NMDA and AMPA receptors, calcium influx, stimulation of the stress-inducible MAPK, p38, and finally collapse of the actin cytoskeleton, retraction of dendritic spines, and a decrease in excitatory neurotransmission (Fig 13). This work provides new insights into the mechanisms of synaptic degeneration in prion diseases, it identifies novel molecular targets for treatment of these disorders, and it allows comparison with pathologic mechanisms operative in other neurodegenerative disorders such as Alzheimer’s disease.

While our previous study focused on retraction of dendritic spines as the main morphological consequence of PrP^Sc^ toxicity [23], the results presented here provide a more complete picture of the underling cellular events. We have shown that PrP^Sc^ exhibits striking functional and morphological specificity in its synaptotoxic effects: it damages excitatory and not inhibitory synapses, and it targets postsynaptic and not presynaptic sites. This selectivity may reflect the presence on dendritic spines of glutamate receptors capable of initiating excitotoxic processes (see below), or the presence of a higher concentration of specific intracellular signaling molecules in these locations. We have also shown that spine loss is accompanied by a reduction in postsynaptic AMPA receptors, and by a decrement in mEPSC frequency and amplitude, suggesting that functional changes in synaptic transmission are early indicators of prion neurotoxicity. Although many studies have emphasized neuronal death as an important feature of prion and other neurodegenerative diseases, our results emphasize the importance of looking specifically at synaptic dysfunction and loss in order to understand the earliest events in the pathological process.

Our evidence suggests that synaptotoxic signaling induced by PrP^Sc^ is initiated by its interaction with PrP^C^ on the cell surface (Fig 13). All of the synaptotoxic effects of PrP^Sc^ observed in our system require expression of endogenous PrP^C^ by the target neurons [this work and 23]. Moreover, neurons from transgenic mice expressing N-terminally deleted forms of PrP^C^ (Δ23–31 and Δ23–111) are resistant to PrP^Sc^ toxicity, pinpointing an essential role for these residues in the conversion and/or signal-transduction processes [23]. The requirement for cell-surface PrP^C^ in our system is consistent with the documented role of PrP^C^ in mediating the neurotoxic effects of PrP^Sc^ *in vivo* [8, 9]. We have hypothesized that neurotoxic signals may be generated either by the initial binding of PrP^Sc^ to PrP^C^, or by the subsequent conversion of PrP^C^ into nascent PrP^Sc^ on the cell surface [23]. Since both PrP^C^ and PrP^Sc^ are attached to the plasma membrane by a GPI anchor, production of intracellular signals would presumably require their interaction with partners that are transmembrane proteins, for example ion channels [see below and 56, 60], adhesion molecules [61], or receptors [62].

We have shown that activation of NMDA and AMPA receptors is required for PrP^Sc^-induced synaptotoxicity in our system, and that specific blockers of these channels prevent dendritic spine retraction. Moreover, PrP^Sc^ induces a rapid (within 30 min) increase in intracellular calcium, mediated by NMDA receptors. These results suggest that glutamate receptor activation accompanied by calcium entry is an early step in the signaling cascade initiated by PrP^Sc^, and that glutamate-induced excitotoxicity may play a role in the ensuing synaptic damage. PrP^C^ has been suggested to be a modulator of NMDA receptor function [63] and posttranslational modification [64], and to interact physically with NMDA receptor subunits [65], processes that could be altered by binding of PrP^Sc^ to cell-surface PrP^C^ during the initial phase of the conversion process. Also, activation of NMDA-dependent transcriptional pathways is an early event during the pre-clinical phase of prion infection in mice, as determined by microarray profiling [66]. More generally, NMDA receptors play an important role in learning and memory [25], and they are known to be involved in many neurodegenerative diseases in the context of excitotoxic activation [14]. Of note, glutamate stimulation of cultured hippocampal neurons causes dendritic abnormalities (spine retraction) and calcium influx that are similar to those seen in our system with PrP^Sc^ treatment [67].

Our results implicate p38 MAPK, specifically the α isoform, in PrP^Sc^-induced synaptotoxicity, based on blockade of dendritic spine retraction and mEPSC reduction by specific inhibitors of this kinase, and by expression of a dominant-negative form of the kinase. Moreover, using immunofluorescence staining, we demonstrated that PrP^Sc^ induces rapid (within 1 hr) phosphorylation of p38 MAPK in dendritic spines, a region where this kinase was previously shown to reside [68]. Thus, PrP^Sc^ activates a localized, p38-mediated signal transduction cascade that precedes dendritic spine retraction. p38 MAPK is, like its counterpart JNK, a stress-activated protein kinase, and it was originally found to be stimulated by a variety of environmental stresses and cytokines that induce inflammation [31, 37]. It has now been implicated in a wide range of functions, including regulation of the cell cycle, induction of cell death, differentiation, and senescence. In the nervous system, p38 MAPK has been found to play a role in neuronal damage and survival, as well as in synaptic plasticity, and it has been linked to a number of neurodegenerative diseases [32, 69]. Consistent with the results presented here, p38 MAPK, and its downstream substrates MK2/3, have been shown to regulate AMPA receptor trafficking, dendritic spine morphology, and synaptic transmission [70]. We have identified MK2/3 as likely substrates for p38 MAPK in the PrP^Sc^ synaptotoxic pathway, based on the use of specific inhibitors, but the identity of the upstream kinases (MAPKKs and MAPKKKs) responsible for p38 MAPK activation remain to be determined. Activation of MAPK pathways typically leads to programmed changes in gene expression [31], and it will be of great interest to characterize these in order to understand PrP^Sc^ synaptotoxicity at a genomic level.

Our results demonstrate that re-organization of the actin cytoskeleton within dendritic spines represents a key cellular correlate of PrP^Sc^ synaptotoxicity, based on the ability of actin stabilizers (SiR-actin) to block the morphological and electrophysiological consequences of PrP^Sc^ treatment. Actin plays an important role in the structure and function of dendritic spines [42]. The equilibrium between G- and F-actin in spines is regulated in an activity-dependent manner, and actin dynamics influences spine development and synaptic plasticity [43, 44]. Postsynaptic receptors, including NMDA and AMPA receptors [71], regulate the actin cytoskeleton via their effect on cytoplasmic actin-binding proteins, either by directly interacting with these proteins or, in the case of NMDA receptors, by increasing intracellular calcium [72, 73]. This mechanism provides a plausible link between PrP^Sc^ activation of NMDA receptors, with rapid influx of calcium, and the resulting abnormalities in dendritic spine morphology and function. There is evidence that p38 MAPK can also influence actin cytoskeletal dynamics [74], providing another potential pathway by which PrP^Sc^ could alter spine morphology via activation of this kinase.

Several previous studies have implicated specific signal transduction pathways in the pathogenesis of prion diseases, including the UPR [46], oxidative stress [75], MAPKs [76–78], phosphoinositide-dependent kinase-1 (PDK1) [79], metabotropic glutamate receptors [80], NMDA receptors [81], and voltage-gated calcium channels [82]. Given published evidence that the PERK/eIF2α arm of the UPR is activated during prion infection, and that pharmacological blockage of this response has therapeutic benefit [46–48], we tested inhibitors of this pathway in our system. However, we found that these inhibitors had no effect on PrP^Sc^-induced loss of dendritic spines. This discrepancy is likely to reflect fundamental differences between the experimental systems employed. All of the previously quoted studies utilized brain slices or tissue samples from human patients or transgenic mice undergoing neurodegenerative changes after prion infection, making it difficult to isolate the primary synaptotoxic mechanisms engaged by PrP^Sc^. In contrast, we have employed a neuronal culture system that allows us to characterize acute responses to exogenous PrP^Sc^ exposure. We have shown that, in this system, treatment with PrP^Sc^ causes a detectable increase in intracellular calcium within 30 min, followed by alterations in electrophysiological properties, with complete collapse of dendritic spines by 24 hrs. Moreover, our experiments have demonstrated the involvement of MAPK signaling cascades, which are typically activated within 60 min of exposure to an extracellular stimulus. Our results do not rule out the engagement of additional pathways, which contribute to pathological changes occurring at later times, once PrP^Sc^ begins to accumulate.

Our results identify novel molecular targets for therapeutic intervention. Previous efforts to develop anti-prion therapies have focused on strategies for inhibiting formation of PrP^Sc^ or enhancing its clearance [83]. In contrast, our studies suggest the possibility of interfering with neurotoxic pathways that lie downstream of PrP^Sc^. We have shown that several kinds of pharmacologic agents prevent PrP^Sc^-induced synaptotoxic effects in our system, including NMDA and AMPA receptor antagonists, and p38 MAPK inhibitors. Importantly, we have found that treatment with a p38 MAPK inhibitor is able to reverse dendritic spine retraction that has already occurred during an initial exposure to PrP^Sc^. This result, which presumably reflects the dynamic nature of dendritic spines, suggests the existence of a therapeutic window for treatment of patients who have already been infected with prions and who might even have sustained a certain level of synaptic damage. The advent of methods for pre-mortem diagnosis of prion diseases [84, 85] makes this type of treatment modality especially compelling. The NMDA receptor antagonist memantine is now a widely used treatment for Alzheimer’s disease [86], and p38 MAP kinase inhibitors have been developed for therapy of inflammatory diseases [87] and CNS disorders, including Alzheimer’s disease [88–90]. It may be feasible to re-purpose these agents for treatment of prion diseases.

It has been proposed that PrP^C^ acts as a cell-surface receptor for Aβ oligomers in Alzheimer’s disease, and that it mediates the neurotoxic effects of these oligomers [51–59]. We have been able to use our neuronal culture system to test whether PrP^Sc^ and Aβ oligomers activate a common synaptotoxic pathway upon binding to PrP^C^. Our results indicate that the two pathways diverge, based on our observation that the synaptotoxic effects of PrP^Sc^ are blocked by p38 MAPK inhibitors but not mGluR5 inhibitors, while the reverse is true for the synaptotoxic effects of Aβ oligomers. The precise differences between the PrP^Sc^ and Aβ synaptotoxic pathways remain to be determined. Our system could be used to probe the synaptotoxic mechanisms activated by other oligomeric aggregates, including tau and α-synuclein, which are thought to participate in an extracellular transmission phase [91], and which may also utilize PrP^C^ as a cell-surface receptor [92].

Chronic neurodegenerative disorders, like prion, Alzheimer’s and Parkinson’s diseases, are likely to involve multiple pathogenic mechanisms, each of which may be operative at different stages of the disease process. The experimental system described here has allowed us to isolate one very early event (synaptic degeneration) in the pathological cascade, and study its cellular and molecular underpinnings. Future experiments will be aimed at translating these findings into animal models and testing the efficacy of therapeutic interventions directed at specific steps in the signaling pathway we have identified.

## Materials and methods

### Low-density neuronal cultures (used for dendritic spine measurements)

Timed-pregnant C57BL/6 mice (referred to as wild-type, WT) were purchased from the Jackson Laboratory (Bar Harbor, ME). *Prnp*^0/0^ mice [7] on a C57BL6 background were obtained from the European Mouse Mutant Archive (EMMA; Rome, Italy), and were maintained in a homozygous state by interbreeding.

Mice carrying the p38AF dominant-negative mutation [35] on a C57BL6 background were obtained from the Jackson Laboratory (B6.Cg-Mapk14^tm1.1Dvb^/J; stock #012736). The mutant allele was maintained in a heterozygous state by breeding with C57BL6 inbred mice. PCR genotyping of tail DNA was performed as per information and protocols are provided by Jackson Laboratory using the following primers: 5’-TAG AGC CAG CCC CAC TTT AGT C-3’ and 5’-GAA GAT GGA TTT TAA GCA TCC GT-3’. The expected PCR products included a 328 bp band representing the dominant-negative allele, and a 195 bp band representing the WT allele.

All procedures involving animals were conducted according to the United States Department of Agriculture Animal Welfare Act and the National Institutes of Health Policy on Humane Care and Use of Laboratory Animals.

Hippocampal neurons were cultured from P0 pups as described [93]. All experiments shown, except those indicated in Figs 1 and 4, were performed with neurons from WT mice. Neurons were seeded at 75 cells/mm^2^ on poly-L-lysine-treated coverslips, and after several hrs the coverslips were inverted onto an astrocyte feeder layer and maintained in NB/B27 medium until used. The astrocyte feeder layer was generated using murine neural stem cells, as described [94]. Neurons were kept in culture for 18–21 days prior to PrP^Sc^ or ADDL treatment.

### Dendritic spine quantitation

Hippocampal neurons cultured as described above were treated with purified PrP^Sc^, ADDLs, or control preparations for 24 hrs, followed by fixation in 4% paraformaldehyde and staining with either Alexa 488-phalloidin or rhodamine-phalloidin (ThermoFischer Scientific, Waltham, MA) to visualize dendritic spines, and anti-tubulin antibodies (Sigma-Aldrich, St. Louis, MO) to visualize axons and dendrites. Images were acquired using a Zeiss 880 (Figs 8 and 9) Zeiss 700 (all other figures) confocal microscope with a 63x objective (N.A. = 1.4). The number of dendritic spines was determined using ImageJ software. Briefly, 2–3 isolated dendritic segments were chosen from each image, and the images adjusted using a threshold that had been optimized to include the outline of the spines but not non-specific signals [95]. The number of spines was normalized to the measured length of the dendritic segment to give the number of spines/μm. For each experiment, 15–24 neurons from 3–4 individual experiments were imaged and quantitated.

Immunostaining was performed with the following primary antibodies and corresponding secondary antibodies: anti-gephyrin (Synaptic Systems, Woodland, CA; cat 147011, 1:500); anti-tau (Santa Cruz Biotechnology, Santa Cruz, CA; cat. Sc5587, 1:500); anti-GluR1 (Abcam, Cambridge, MA; cat. Ab31232, 1:500); anti-synaptophysin (Millipore Sigma, St Louis, MO; cat. S5768, 1:500). Quantitation of gephyrin, GluR1, and synaptophysin staining was performed using ImageJ to count the number of fluorescent puncta per μm along isolated dendritic segments (similar to the method described above to quantitate dendritic spine numbers after phalloidin staining).

### Calcium imaging

Hippocampal neurons cultured as described above were washed once with PBS before treatment with Fluo-3 (ThermoFisher Scientific, Waltham, MA) at final concentration of 5 μM. Cells were incubated for 20–30 min at 37°C, followed by a several washes to remove extracellular dye. Neurons were imaged in the green fluorescence channel of an Olympus wide field microscope with 20X objective. Each neuron was imaged for 5 min before either drug or solvent control was added, after which imaging was continued for an additional 30 min. The average signal during the 5 min pre-treatment was considered to represent the baseline calcium level for each cell. The proportion of 30 min recording period during which the calcium signal was ≥ 2X the baseline level was calculated and used as a measure of net calcium accumulation.

### Measurement of p38 MAPK phosphorylation in dendritic spines

Low-density hippocampal neuronal cultures were treated with purified PrP^Sc^ or mock preparations for 1 or 24 hrs, followed by fixation in 4% paraformaldehyde containing phosphatase inhibitors (Roche-Sigma, 04 906 845 001) and permeabilization in 1% Triton X-100 for 5 min. Dual immunostaining was performed with anti-phospho-p38 antibody (Thr180/Tyr182) (Cell Signaling, mAb #4511S, 1:100) and anti-total p38 antibody (Abcam, ab31828, 1:300), followed by Alexa 546 and Alexa 633 secondary antibodies, respectively. Cultures were also stained with Alexa 488-phalloidin to visualize dendritic spines. Multi-stack images were acquired using a Zeiss 880 confocal microscope with a 63x objective (N.A. = 1.4). The fluorescence intensities of the phospho-p38 and total p38 signals were quantitated within a region of interest (ROI) using ImageJ software. Images of phalloidin staining were used to determine the location of intact or collapsed dendritic spines. Two or three isolated ROIs on each neuron, coinciding with locations of individual dendritic spines, were captured at 10X zoom, and then the fluorescence intensity and area were determined within the ROI. The fluorescence intensities were normalized to the area of the ROI, and the ratio of phospho-p38/total p38 staining was calculated. For each experiment, 10–12 neurons from 3–4 individual experiments were analyzed.

### Electrophysiological analysis using mixed hippocampal/glial cultures

With the exception of the experiment shown in S1 Fig, hippocampal cultures used for electrophysiological recording were prepared using a procedure that differs from the one used to prepare cultures for dendritic spine imaging. Briefly, hippocampi from newborn pups of the indicated genotypes were dissected and treated with 0.25% trypsin at 37°C for 12 min [96]. Cells were plated at a density of 65,000 cells/cm^2^ on poly-D-lysine-coated coverslips in DMEM medium with 10% F12 and 10% FBS.

Recordings were made from hippocampal neurons cultured for 18–20 days and treated for 24 hrs with purified PrP^Sc^ or control preparations. Whole-cell patch clamp recordings were collected using standard techniques. Pipettes were pulled from borosilicate glass and polished to an open resistance of 2–5 megaohms. Experiments were conducted at room temperature with the following solutions: internal, 140 mM Cs-glucuronate, 5 mM CsCl, 4 mM MgATP, 1 mM Na_2_GTP, 10 mM EGTA, and 10 mM HEPES (pH 7.4 with CsOH); external, 150 mM NaCl, 4 mM KCl, 2 mM CaCl_2_, 2 mM MgCl_2_, 10 mM glucose, and 10 mM HEPES (pH 7.4 with NaOH). Current signals were collected from a Multiclamp 700B amplifier (Molecular Devices, Sunnyvale, CA), digitized with a Digidata 1550A interface (Axon Instruments, Union City, CA), and saved to disc for analysis with PClamp 10 software. Miniature excitatory postsynaptic currents (mEPSCs) were recorded in the presence of TTX (1 μM, Abcam, Cat. # ab120054) and picrotoxin (100 μM, Abcam, Cat. # ab120315). Miniature inhibitory postsynaptic currents (mIPSCs) were recorded in the presence of TTX (1 μM) and CNQX (20 μM, Abcam, Cat. # ab120044). Frequencies and amplitudes of the mEPSCs and mIPSCs were quantitated by Clampfit (Molecular Devices, CA).

### Purification of PrP^Sc^

Purification was carried out as previously described [23, 97]. In a typical preparation (used for all experiments, except those shown Figs 8 and 9), 18 RML-infected C57BL6 brains were homogenized in 3 ml of 10% sarkosyl in TEND (10 mM Tris-HCl [pH 8], 1 mM EDTA, 130 mM NaCl, and 1 mM dithiothreitol) containing Complete Protease Inhibitor Cocktail (Roche Diagnostics, cat. no. 11836153001) using a glass bead homogenizer. Brain homogenates were incubated on ice for 1 hr and centrifuged at 22,000 x g for 30 min at 4°C. The supernatant was kept on ice, while the pellet was resuspended in 1 ml of 10% sarkosyl in TEND, incubated for 1 hr on ice, and then centrifuged at 22,000 x g for 30 min at 4°C. The pellet was discarded while the supernatants were pooled and centrifuged at 150,000 x g for 2.5 h at 4°C. The new supernatants were discarded, while the pellets were rinsed with 50 ml of 100 mM NaCl, 1% sulfobetaine (SB) 3–14 in TEND plus protease inhibitors, and then pooled by resuspending them in 1 ml of the wash buffer, and centrifuging at 180,000 x g for 2 hr at 20°C. The supernatant was discarded, and the pellet was rinsed with 50 ml of TMS (10 mM Tris-HCl [pH 7.0], 5 mM MgCl_2_, and 100 mM NaCl) plus protease inhibitors, resuspended in 600 μl of the same buffer containing 100 mg/ml RNase A and incubated for 2 hr at 37°C. The sample was then incubated with 5 mM CaCl_2_, 20 mg/ml DNase I for 2 hr at 37°C. To stop the enzymatic digestion, EDTA was added to a final concentration of 20 mM, and the sample was mixed with an equal volume of TMS containing 1% SB 3–14. The sample was gently deposited on a 100 μl cushion of 1 M sucrose, 100 mM NaCl, 0.5% SB 3–14, and 10 mM Tris-HCl (pH 7.4), and centrifuged at 180,000 x g for 2 hr at 4°C. The supernatant was discarded and the pellet was rinsed with 50 μl of 0.5% SB 3–14 in PBS, resuspended in 1 ml of the same buffer, subjected to 5 X 5 sec pulses of bath sonication with a Bandelin Sonopuls Ultrasonicator (Amtrex Technologies, Montreal, Canada) at 90% power, and centrifuged at 180,000 x g for 15 min at 4°C. The final supernatant was discarded and the final pellet was resuspended in 900 μl of PBS (50 μl for each starting brain) and sonicated 5 times for 5 sec. Aliquots were stored at -80°C. Mock purifications were also carried out from age-match, uninfected brains. The purified preparations were evaluated by SDS-PAGE followed by silver staining and Western blotting.

For the experiments shown in Figs 8 and 9, PrP^Sc^ was purified using the pronase E method [23].

In all experiments, purified PrP^Sc^ was added to neuronal cultures at a final concentration of 4.4 μg/ml, identical to what was used in our previous study [23]. An equivalent amount of mock material was used, based on purification from the same proportion of brain tissue.

### ScN2a cell assay

The uninfected Neuro-2a cells (N2a) were from the ATCC (Cat. #: ATCC CCL-131). A scrapie-susceptible sub-clone (N2a.3) infected with RML prions [98] were plated in 6-well plates. Cells were treated for 3 days with test compounds (p38 MAPK and MK2/3/5 inhibitors), or with DMSO vehicle as a negative control and Congo red as a positive control. Cultures were then split at a 1:5 ratio and treatment with compounds in fresh medium continued for 4 more days. At the end of the 7-day treatment, cells were lysed in 300 μl of lysis buffer (0.5% NP-40, 0.5% deoxycholate, 10mM Tris-HCl, pH8 and 100mM NaCl) and protein concentration was measured using BCA assay (ThermoFisher Scientific, Waltham, MA). Samples were then treated with proteinase K (40 μg/ml) at 37°C for 1 hr. Digestion was stopped by addition of 10x Complete Protease Inhibitor Cocktail (Roche, Indianapolis, IN). Samples were centrifuged at 180,000 x g for 1 hr at 4°C. Pellets were dissolved in 20 μl of 1x Laemmli loading buffer (Bio-Rad, Hercules, CA) and were boiled for 3 min before loading on pre-cast 12% SDS-PAGE Criterion gels (Bio-Rad, Hercules, CA). Western blotting was performed according to standard procedures. PrP^Sc^ was detected using the anti-prion antibody D18 [99] and HRP-coupled, anti-human secondary antibody (Jackson ImmnoResearch, West Grove, PA). Quantitation was performed using the ImageJ gel quantification function.

### ADDL preparation

ADDLs were prepared from synthetic Aβ 1–42 peptide as previously described [55, 100]. Aβ peptide (ERI Amyloid Laboratory, Oxford, CT) was dissolved in HFIP at a concentration of 1 mM and sonicated for 10 min in an ice-water bath. The sample was allowed to incubate at room temperature for 1 hr before it was transferred to a centrifuge tube and spun at 15,800 x g for 1 min. The supernatant was transferred into a new glass vial and was dried under a fume hood with nitrogen gas. The film of dried peptide was dissolved in DMSO, and then diluted into Ham’s F12 phenol-red-free medium (Gibco/ThermoFisher scientific, Waltham, MA) to a final concentration of 100 μM. The sample was incubated at room temperature for 16 hrs and was then centrifuged for 15 min at 15,800 x g. The supernatant was aliquoted, flash-frozen in liquid nitrogen, and stored at -80°C.

### Ethics statement

All procedures involving animals were conducted according to the United States Department of Agriculture Animal Welfare Act and the National Institutes of Health Policy on Humane Care and Use of Laboratory Animals. Ethical approval (AN-14997) was obtained from Boston University medical center institutional animal care and use committee.

## Supporting information

S1 Fig The PrP^Sc^ effect on mEPSCs is comparable in two types of neuronal culture systems. Hippocampal neurons were cultured on coverslips at low-density over an astrocyte feeder layer, the same procedure used for visualization of dendritic spines. Cultures were treated for 24 hrs with either purified PrP^Sc^ or with mock-purified material, after which mEPSCs were recorded and their frequencies (A) and amplitudes (B) quantitated. N = 15 from 2 independent experiments. ***p<0.001 by Student’s t-test.
(TIF)

S2 Fig PrP^Sc^ does not affect inhibitory postsynaptic markers. Hippocampal neurons were treated for 24 hrs with mock-purified material (A, B) or with purified PrP^Sc^ (C, D). Cultures were then fixed and stained with fluorescent phalloidin (green) (A, C) along with an antibody to the inhibitory postsynaptic marker, gephyrin (red) (B, D). Quantitation of spine number and gephyrin staining is shown in panel E, normalized to the values in mock-treated cultures. Pooled measurements were collected from 15–20 cells from 3 independent experiments. ***p<0.001 by Student’s t-test; N.S., not significantly different. Scale bar in panel D = 20 μm (also applicable to panels A-C).
(TIF)

S3 Fig Voltage-gated calcium channels do not play a major role in PrP^Sc^ synaptotoxicity. Hippocampal neurons were treated for 24 hrs with purified PrP^Sc^ in the presence or absence of inhibitors of R-, T-, N-, P/Q- and L-type voltage-gated calcium channels (VGCCs) (bars labeled Plus PrP^Sc^). A parallel set of cultures was treated with inhibitor without PrP^Sc^ (bars labeled Minus PrP^Sc^). The bar labeled Mock represents cultures treated with mock-purified material in the absence of inhibitors. Pooled measurements of spine number were collected from 15–20 cells from 3 independent experiments. *p<0.05; ***p<0.001 by Student’s t-test; N.S., not significantly different. The inhibitors used are listed in Table 1.
(TIF)

S4 Fig The α isoform of p38 MAPK plays an essential role in PrP^Sc^ synaptotoxicity. Hippocampal neurons were treated for 24 hrs with mock-purified material (A), purified PrP^Sc^ (B), or purified PrP^Sc^ in the presence of a p38α MAPK inhibitor (VX745, 100 nM) (C). Dendritic spines were then visualized by fluorescent phalloidin staining (A-C). Pooled measurements of spine number were collected from 15–20 cells from 3 independent experiments (D). The bar labeled p38αi represents cultures treated with inhibitor without PrP^Sc^. Parallel cultures were analyzed by patch clamping to measure mEPSC frequency and amplitude (E-G).). N = 10 cells from 2 independent experiments. ***p<0.001 and * p<0.05 by Student’s t-test; N.S., not significantly different. Scale bar in panel C = 20 μm (also applicable to panels A and B).
(TIF)

S5 Fig p38 MAPK and MK inhibitors do not affect PrP^Sc^ propagation in ScN2a cells. ScN2a cells were treated for 3 days with DMSO vehicle, Congo red (5 μm), p38 MAPK inhibitor (SB239063, 10 μM), or MK2/3/5 inhibitor (CAS1186648, 500 nM), after which cells were split at a 1:5 ratio and fresh inhibitors were added for 4 more days. At the end of the 7-day treatment, cells were harvested and lysed. BCA protein assays of lysates were performed as a measure of drug cytotoxicity (A). Cell lysates were also subjected to proteinase K digestion followed by Western blotting to reveal proteinase K-resistant PrP^Sc^ (B). ***p<0.001 by Student’s t-test; N.S., not significantly different. Data were derived from triplicate cultures.
(TIF)

S6 Fig The unfolded protein response does not play a major role in PrP^Sc^ synaptotoxicity. Hippocampal neurons from WT mice were treated for 24 hr with integrated stress response inhibitor (Trans-ISRIB, 20 nM) alone (A), PERK inhibitor (GSK2606414, 500 nM) alone (B), or with the respective inhibitors in combination with purified PrP^Sc^ (C, D). Neurons were then fixed and stained with fluorescent phalloidin. Pooled measurements of dendritic spine number were collected from 15–20 cells from 3 independent experiments (E). *p<0.05 by Student’s t-test; N.S., not significantly different. Scale bar in panel D = 20 μm (also applicable to panels A-C).
(TIF)

S7 Fig Aβ oligomers cause PrP^C^-dependent dendritic spine retraction. Primary hippocampal neurons from wild-type (WT) mice (A, B) or PrP knockout mice (*Prn-p*^0/0^) (C, D) were treated for 24 hrs with vehicle (A, C) or ADDLs (1.6 μM) (B, D). Neurons were then fixed and stained with fluorescent phalloidin (green) to visualize F-actin in dendritic spines, and with anti-tubulin (red) to visualize overall dendritic morphology. Pooled measurements of dendritic spine number were collected from 15–20 cells from 3 indepdendent experiments (E). ***p<0.001 by Student’s t-test; N.S., not significantly different. Scale bar in panel D = 20 μm (also applicable to panels A-C).
(TIF)

## References

[1] Prusiner SB. Prions. Proc Natl Acad Sci USA. 1998;95:13363–83. 9811807 10.1073/pnas.95.23.13363 PMC33918

[2] Aguzzi A, Polymenidou M. Mammalian prion biology: one century of evolving concepts. Cell. 2004;116(2):313–27. . 14744440 10.1016/s0092-8674(03)01031-6

[3] Kocisko DA, Come JH, Priola SA, Chesebro B, Raymond GJ, Lansbury PT, et al Cell-free formation of protease-resistant prion protein. Nature. 1994;370:471–4. 10.1038/370471a0 7913989

[4] Bessen RA, Kocisko DA, Raymond GJ, Nandan S, Lansbury PT, Caughey B. Non-genetic propagation of strain-specific properties of scrapie prion protein. Nature. 1995;375(6533):698–700. 10.1038/375698a0 7791905

[5] Büeler H, Aguzzi A, Sailer A, Greiner RA, Autenried P, Aguet M, et al Mice devoid of PrP are resistant to scrapie. Cell. 1993;73(7):1339–47. 8100741 10.1016/0092-8674(93)90360-3

[6] Prusiner SB, Groth D, Serban A, Koehler R, Foster D, Torchia M, et al Ablation of the prion protein (PrP) gene in mice prevents scrapie and facilitates production of anti-PrP antibodies. Proc Natl Acad Sci USA. 1993;90(22):10608–12. Epub 1993/11/15. ; PubMed Central PMCID: PMCPMC47826. 7902565 10.1073/pnas.90.22.10608 PMC47826

[7] Büeler H, Fischer M, Lang Y, Fluethmann H, Lipp H-P, DeArmond SJ, et al Normal development and behavior of mice lacking the neuronal cell-surface PrP protein. Nature. 1992;356:577–82. 10.1038/356577a0 1373228

[8] Brandner S, Isenmann S, Raeber A, Fischer M, Sailer A, Kobayashi Y, et al Normal host prion protein necessary for scrapie-induced neurotoxicity. Nature. 1996;379:339–43. 10.1038/379339a0 8552188

[9] Mallucci G, Dickinson A, Linehan J, Klohn PC, Brandner S, Collinge J. Depleting neuronal PrP in prion infection prevents disease and reverses spongiosis. Science. 2003;302(5646):871–4. 10.1126/science.1090187 . 14593181

[10] Sandberg MK, Al-Doujaily H, Sharps B, Clarke AR, Collinge J. Prion propagation and toxicity in vivo occur in two distinct mechanistic phases. Nature. 2011;470(7335):540–2. 10.1038/nature09768 . 21350487

[11] Sandberg MK, Al-Doujaily H, Sharps B, De Oliveira MW, Schmidt C, Richard-Londt A, et al Prion neuropathology follows the accumulation of alternate prion protein isoforms after infective titre has peaked. Nat Commun. 2014;5:4347 10.1038/ncomms5347 ; PubMed Central PMCID: PMC4104459. 25005024 PMC4104459

[12] Biasini E, Turnbaugh JA, Unterberger U, Harris DA. Prion protein at the crossroads of physiology and disease. Trends Neurosci. 2012;35(2):92–103. Epub 2011/12/06. doi: S0166-2236(11)00173-1 [pii] 10.1016/j.tins.2011.10.002 ; PubMed Central PMCID: PMC3273588. 22137337 PMC3273588

[13] Henstridge CM, Pickett E, Spires-Jones TL. Synaptic pathology: A shared mechanism in neurological disease. Ageing Res Rev. 2016;28:72–84. Epub 2016/04/25. 10.1016/j.arr.2016.04.005 . 27108053

[14] Herms J, Dorostkar MM. Dendritic spine pathology in neurodegenerative diseases. Annu Rev Pathol. 2016;11:221–50. Epub 2016/02/26. 10.1146/annurev-pathol-012615-044216 . 26907528

[15] Belichenko PV, Brown D, Jeffrey M, Fraser JR. Dendritic and synaptic alterations of hippocampal pyramidal neurones in scrapie-infected mice. Neuropathol Appl Neurobiol. 2000;26(2):143–9. . 10840277 10.1046/j.1365-2990.2000.026002143.x

[16] Campeau JL, Wu G, Bell JR, Rasmussen J, Sim VL. Early increase and late decrease of purkinje cell dendritic spine density in prion-infected organotypic mouse cerebellar cultures. PLoS One. 2013;8(12):e81776 10.1371/journal.pone.0081776 ; PubMed Central PMCID: PMC3847088. 24312586 PMC3847088

[17] Clinton J, Forsyth C, Royston MC, Roberts GW. Synaptic degeneration is the primary neuropathological feature in prion disease: a preliminary study. Neuroreport. 1993;4(1):65–8. . 8453038 10.1097/00001756-199301000-00017

[18] Cunningham C, Deacon R, Wells H, Boche D, Waters S, Diniz CP, et al Synaptic changes characterize early behavioural signs in the ME7 model of murine prion disease. Eur J Neurosci. 2003;17(10):2147–55. . 12786981 10.1046/j.1460-9568.2003.02662.x

[19] Jeffrey M, Halliday WG, Bell J, Johnston AR, MacLeod NK, Ingham C, et al Synapse loss associated with abnormal PrP precedes neuronal degeneration in the scrapie-infected murine hippocampus. Neuropathol Appl Neurobiol. 2000;26(1):41–54. . 10736066 10.1046/j.1365-2990.2000.00216.x

[20] Kovacs GG, Preusser M, Strohschneider M, Budka H. Subcellular localization of disease-associated prion protein in the human brain. Am J Pathol. 2005;166(1):287–94. 10.1016/S0002-9440(10)62252-3 . 15632020 PMC1602295

[21] Fuhrmann M, Mitteregger G, Kretzschmar H, Herms J. Dendritic pathology in prion disease starts at the synaptic spine. J Neurosci. 2007;27(23):6224–33. 10.1523/JNEUROSCI.5062-06.2007 . 17553995 PMC6672160

[22] Mallucci GR. Prion neurodegeneration: starts and stops at the synapse. Prion. 2009;3(4):195–201. ; PubMed Central PMCID: PMC2807691. 19887910 10.4161/pri.3.4.9981 PMC2807691

[23] Fang C, Imberdis T, Garza MC, Wille H, Harris DA. A neuronal culture system to detect prion synaptotoxicity. PLoS Pathog. 2016;12(5):e1005623 10.1371/journal.ppat.1005623 ; PubMed Central PMCID: PMCPMC4881977. 27227882 PMC4881977

[24] Nimchinsky EA, Sabatini BL, Svoboda K. Structure and function of dendritic spines. Annu Rev Physiol. 2002;64:313–53. 10.1146/annurev.physiol.64.081501.160008 . 11826272

[25] Sala C, Segal M. Dendritic spines: the locus of structural and functional plasticity. Physiol Rev. 2014;94(1):141–88. 10.1152/physrev.00012.2013 . 24382885

[26] Hynd MR, Scott HL, Dodd PR. Glutamate-mediated excitotoxicity and neurodegeneration in Alzheimer's disease. Neurochem Int. 2004;45(5):583–95. 10.1016/j.neuint.2004.03.007 . 15234100

[27] King AE, Woodhouse A, Kirkcaldie MT, Vickers JC. Excitotoxicity in ALS: Overstimulation, or overreaction? Exp Neurol. 2016;275 Pt 1:162–71. 10.1016/j.expneurol.2015.09.019 . 26584004

[28] Ambrosi G, Cerri S, Blandini F. A further update on the role of excitotoxicity in the pathogenesis of Parkinson's disease. J Neural Transm. 2014;121(8):849–59. Epub 2014/01/02. 10.1007/s00702-013-1149-z . 24380931

[29] Sepers MD, Raymond LA. Mechanisms of synaptic dysfunction and excitotoxicity in Huntington's disease. Drug Discov Today. 2014;19(7):990–6. 10.1016/j.drudis.2014.02.006 . 24603212

[30] Lewerenz J, Maher P. Chronic glutamate toxicity in neurodegenerative diseases-What is the evidence? Front Neurosci. 2015;9:469 Epub 2016/01/07. 10.3389/fnins.2015.00469 ; PubMed Central PMCID: PMCPMC4679930. 26733784 PMC4679930

[31] Morrison DK. MAP kinase pathways. Cold Spring Harb Perspect Biol. 2012;4(11): pii: a011254. 10.1101/cshperspect.a011254 ; PubMed Central PMCID: PMCPMC3536342. 23125017 PMC3536342

[32] Correa SA, Eales KL. The role of p38 MAPK and its substrates in neuronal plasticity and neurodegenerative disease. J Signal Transduct. 2012;2012:649079 Epub 2012/07/14. 10.1155/2012/649079 ; PubMed Central PMCID: PMCPMC3389708. 22792454 PMC3389708

[33] Zarubin T, Han J. Activation and signaling of the p38 MAP kinase pathway. Cell Res. 2005;15(1):11–8. 10.1038/sj.cr.7290257 . 15686620

[34] Cuenda A, Cohen P, Buee-Scherrer V, Goedert M. Activation of stress-activated protein kinase-3 (SAPK3) by cytokines and cellular stresses is mediated via SAPKK3 (MKK6); comparison of the specificities of SAPK3 and SAPK2 (RK/p38). EMBO J. 1997;16(2):295–305. 10.1093/emboj/16.2.295 ; PubMed Central PMCID: PMC1169636. 9029150 PMC1169636

[35] Wong ES, Le Guezennec X, Demidov ON, Marshall NT, Wang ST, Krishnamurthy J, et al p38MAPK controls expression of multiple cell cycle inhibitors and islet proliferation with advancing age. Dev Cell. 2009;17(1):142–9. Epub 2009/07/22. 10.1016/j.devcel.2009.05.009 . 19619499

[36] Adams RH, Porras A, Alonso G, Jones M, Vintersten K, Panelli S, et al Essential role of p38alpha MAP kinase in placental but not embryonic cardiovascular development. Mol Cell. 2000;6(1):109–16. Epub 2000/08/19. . 10949032

[37] Cuadrado A, Nebreda AR. Mechanisms and functions of p38 MAPK signalling. Biochem J. 2010;429(3):403–17. 10.1042/BJ20100323 . 20626350

[38] Shiryaev A, Moens U. Mitogen-activated protein kinase p38 and MK2, MK3 and MK5: menage a trois or menage a quatre? Cell Signal. 2010;22(8):1185–92. Epub 2010/03/17. 10.1016/j.cellsig.2010.03.002 . 20227494

[39] Dingar D, Benoit MJ, Mamarbachi AM, Villeneuve LR, Gillis MA, Grandy S, et al Characterization of the expression and regulation of MK5 in the murine ventricular myocardium. Cell Signal. 2010;22(7):1063–75. 10.1016/j.cellsig.2010.02.009 ; PubMed Central PMCID: PMC3415464. 20214976 PMC3415464

[40] Caughey B, Race RE. Potent inhibition of scrapie-associated PrP accumulation by Congo red. J Neurochem. 1992;59(2):768–71. Epub 1992/08/01. . 1352803 10.1111/j.1471-4159.1992.tb09437.x

[41] Caughey B, Ernst D, Race RE. Congo red inhibition of scrapie agent replication. J Virol. 1993;67:6270–2. 8103804 10.1128/jvi.67.10.6270-6272.1993 PMC238051

[42] Spence EF, Soderling SH. Actin out: regulation of the synaptic cytoskeleton. J Biol Chem. 2015;290(48):28613–22. Epub 2015/10/11. 10.1074/jbc.R115.655118 ; PubMed Central PMCID: PMCPMC4661376. 26453304 PMC4661376

[43] Hlushchenko I, Koskinen M, Hotulainen P. Dendritic spine actin dynamics in neuronal maturation and synaptic plasticity. Cytoskeleton. 2016;73(9):435–41. 10.1002/cm.21280 . 26849484

[44] Hotulainen P, Hoogenraad CC. Actin in dendritic spines: connecting dynamics to function. J Cell Biol. 2010;189(4):619–29. 10.1083/jcb.201003008 ; PubMed Central PMCID: PMC2872912. 20457765 PMC2872912

[45] Lukinavicius G, Reymond L, D'Este E, Masharina A, Gottfert F, Ta H, et al Fluorogenic probes for live-cell imaging of the cytoskeleton. Nat Methods. 2014;11(7):731–3. Epub 2014/05/27. 10.1038/nmeth.2972 . 24859753

[46] Moreno JA, Radford H, Peretti D, Steinert JR, Verity N, Martin MG, et al Sustained translational repression by eIF2alpha-P mediates prion neurodegeneration. Nature. 2012;485(7399):507–11. 10.1038/nature11058 ; PubMed Central PMCID: PMC3378208. 22622579 PMC3378208

[47] Halliday M, Radford H, Sekine Y, Moreno J, Verity N, le Quesne J, et al Partial restoration of protein synthesis rates by the small molecule ISRIB prevents neurodegeneration without pancreatic toxicity. Cell Death Dis. 2015;6:e1672 Epub 2015/03/06. 10.1038/cddis.2015.49 ; PubMed Central PMCID: PMCPMC4385927. 25741597 PMC4385927

[48] Halliday M, Radford H, Zents KAM, Molloy C, Moreno JA, Verity NC, et al Repurposed drugs targeting eIF2α-P-mediated translational repression prevent neurodegeneration in mice. Brain. 2017;140(6):1768–83. Epub 2017/04/22. 10.1093/brain/awx074 ; PubMed Central PMCID: PMCPMC5445255. 28430857 PMC5445255

[49] Sidrauski C, Tsai JC, Kampmann M, Hearn BR, Vedantham P, Jaishankar P, et al Pharmacological dimerization and activation of the exchange factor eIF2B antagonizes the integrated stress response. Elife. 2015;4:e07314 Epub 2015/04/16. 10.7554/eLife.07314 ; PubMed Central PMCID: PMCPMC4426669. 25875391 PMC4426669

[50] Sekine Y, Zyryanova A, Crespillo-Casado A, Fischer PM, Harding HP, Ron D. Stress responses. Mutations in a translation initiation factor identify the target of a memory-enhancing compound. Science. 2015;348(6238):1027–30. Epub 2015/04/11. 10.1126/science.aaa6986 ; PubMed Central PMCID: PMCPMC4538794. 25858979 PMC4538794

[51] Laurén J, Gimbel DA, Nygaard HB, Gilbert JW, Strittmatter SM. Cellular prion protein mediates impairment of synaptic plasticity by amyloid-β oligomers. Nature. 2009;457(7233):1128–32. 10.1038/nature07761 . 19242475 PMC2748841

[52] Fluharty BR, Biasini E, Stravalaci M, Sclip A, Diomede L, Balducci C, et al An N-terminal fragment of the prion protein binds to amyloid-β oligomers and inhibits their neurotoxicity *in vivo*. J Biol Chem. 2013;288:7857–66. Epub 2013/01/31. doi: M112.423954 [pii] 10.1074/jbc.M112.423954 . 23362282 PMC3597823

[53] Chen S, Yadav SP, Surewicz WK. Interaction between human prion protein and amyloid-β (Aβ) oligomers: the role of N-terminal residues. J Biol Chem. 2010;285:26377–83. 10.1074/jbc.M110.145516 . 20576610 PMC2924066

[54] Nicoll AJ, Panico S, Freir DB, Wright D, Terry C, Risse E, et al Amyloid-β nanotubes are associated with prion protein-dependent synaptotoxicity. Nat Commun. 2013;4:2416 10.1038/ncomms3416 . 24022506 PMC3908552

[55] Bove-Fenderson E, Urano R, Straub JE, Harris DA. Cellular prion protein targets amyloid-β fibril ends via its C-terminal domain to prevent elongation. J Biol Chem. 2017;292(41):16858–71. Epub 2017/08/27. 10.1074/jbc.M117.789990 ; PubMed Central PMCID: PMCPMC5641888. 28842494 PMC5641888

[56] Um JW, Kaufman AC, Kostylev M, Heiss JK, Stagi M, Takahashi H, et al Metabotropic glutamate receptor 5 is a coreceptor for Alzheimer Aβ oligomer bound to cellular prion protein. Neuron. 2013;79(5):887–902. 10.1016/j.neuron.2013.06.036 ; PubMed Central PMCID: PMC3768018. 24012003 PMC3768018

[57] Um JW, Nygaard HB, Heiss JK, Kostylev MA, Stagi M, Vortmeyer A, et al Alzheimer amyloid-β oligomer bound to postsynaptic prion protein activates Fyn to impair neurons. Nat Neurosci. 2012;15(9):1227–35. Epub 2012/07/24. 10.1038/nn.3178 [pii]. ; PubMed Central PMCID: PMC3431439. 22820466 PMC3431439

[58] Kaufman AC, Salazar SV, Haas LT, Yang J, Kostylev MA, Jeng AT, et al Fyn inhibition rescues established memory and synapse loss in Alzheimer mice. Ann Neurol. 2015;77(6):953–71. 10.1002/ana.24394 ; PubMed Central PMCID: PMC4447598. 25707991 PMC4447598

[59] Haas LT, Salazar SV, Smith LM, Zhao HR, Cox TO, Herber CS, et al Silent allosteric modulation of mGluR5 maintains glutamate signaling while rescuing Alzheimer's mouse phenotypes. Cell Rep. 2017;20(1):76–88. Epub 2017/07/07. 10.1016/j.celrep.2017.06.023 ; PubMed Central PMCID: PMCPMC5547898. 28683325 PMC5547898

[60] Beraldo FH, Arantes CP, Santos TG, Machado CF, Roffe M, Hajj GN, et al Metabotropic glutamate receptors transduce signals for neurite outgrowth after binding of the prion protein to laminin γ1 chain. FASEB J. 2011;25(1):265–79. Epub 2010/09/30. 10.1096/fj.10-161653 . 20876210

[61] Schmitt-Ulms G, Legname G, Baldwin MA, Ball HL, Bradon N, Bosque PJ, et al Binding of neural cell adhesion molecules (N-CAMs) to the cellular prion protein. J Mol Biol. 2001;314(5):1209–25. 10.1006/jmbi.2000.5183 . 11743735

[62] Taylor DR, Hooper NM. The low-density lipoprotein receptor-related protein 1 (LRP1) mediates the endocytosis of the cellular prion protein. Biochem J. 2007;402(1):17–23. 10.1042/BJ20061736 . 17155929 PMC1783995

[63] Black SA, Stys PK, Zamponi GW, Tsutsui S. Cellular prion protein and NMDA receptor modulation: protecting against excitotoxicity. Front Cell Dev Biol. 2014;2:45 10.3389/fcell.2014.00045 ; PubMed Central PMCID: PMC4207032. 25364752 PMC4207032

[64] Gasperini L, Meneghetti E, Pastore B, Benetti F, Legname G. Prion protein and copper cooperatively protect neurons by modulating NMDA receptor through S-nitrosylation. Antioxid Redox Signal. 2015;22(9):772–84. 10.1089/ars.2014.6032 ; PubMed Central PMCID: PMC4361008. 25490055 PMC4361008

[65] Khosravani H, Zhang Y, Tsutsui S, Hameed S, Altier C, Hamid J, et al Prion protein attenuates excitotoxicity by inhibiting NMDA receptors. J Cell Biol. 2008;181(3):551–65. 10.1083/jcb.200711002 . 18443219 PMC2364707

[66] Majer A, Medina SJ, Niu Y, Abrenica B, Manguiat KJ, Frost KL, et al Early mechanisms of pathobiology are revealed by transcriptional temporal dynamics in hippocampal CA1 neurons of prion infected mice. PLoS Pathog. 2012;8(11):e1003002 Epub 2012/11/13. 10.1371/journal.ppat.1003002 ; PubMed Central PMCID: PMCPMC3493483. 23144617 PMC3493483

[67] Hasbani MJ, Schlief ML, Fisher DA, Goldberg MP. Dendritic spines lost during glutamate receptor activation reemerge at original sites of synaptic contact. J Neurosci. 2001;21(7):2393–403. . 11264313 10.1523/JNEUROSCI.21-07-02393.2001 PMC6762381

[68] Lee SH, Park J, Che Y, Han PL, Lee JK. Constitutive activity and differential localization of p38α and p38β MAPKs in adult mouse brain. J Neurosci Res. 2000;60(5):623–31. Epub 2000/05/23. 10.1002/(SICI)1097-4547(20000601)60:5<623::AID-JNR7>3.0.CO;2-4 . 10820433

[69] Thomas GM, Huganir RL. MAPK cascade signalling and synaptic plasticity. Nat Rev Neurosci. 2004;5(3):173–83. Epub 2004/02/21. 10.1038/nrn1346 . 14976517

[70] Eales KL, Palygin O, O'Loughlin T, Rasooli-Nejad S, Gaestel M, Muller J, et al The MK2/3 cascade regulates AMPAR trafficking and cognitive flexibility. Nat Commun. 2014;5:4701 Epub 2014/08/20. 10.1038/ncomms5701 ; PubMed Central PMCID: PMCPMC4143933. 25134715 PMC4143933

[71] Fischer M, Kaech S, Wagner U, Brinkhaus H, Matus A. Glutamate receptors regulate actin-based plasticity in dendritic spines. Nat Neurosci. 2000;3(9):887–94. 10.1038/78791 . 10966619

[72] Raveendran R, Devi Suma Priya S, Mayadevi M, Steephan M, Santhoshkumar TR, Cheriyan J, et al Phosphorylation status of the NR2B subunit of NMDA receptor regulates its interaction with calcium/calmodulin-dependent protein kinase II. J Neurochem. 2009;110(1):92–105. 10.1111/j.1471-4159.2009.06108.x . 19453375

[73] Wyszynski M, Lin J, Rao A, Nigh E, Beggs AH, Craig AM, et al Competitive binding of alpha-actinin and calmodulin to the NMDA receptor. Nature. 1997;385(6615):439–42. 10.1038/385439a0 . 9009191

[74] Sugiura H, Tanaka H, Yasuda S, Takemiya T, Yamagata K. Transducing neuronal activity into dendritic spine morphology: new roles for p38 MAP kinase and N-cadherin. The Neuroscientist: a review journal bringing neurobiology, neurology and psychiatry. 2009;15(1):90–104. Epub 2009/02/17. 10.1177/1073858408324024 . 19218233

[75] Herrmann US, Sonati T, Falsig J, Reimann RR, Dametto P, O'Connor T, et al Prion infections and anti-PrP antibodies trigger converging neurotoxic pathways. PLoS Pathog. 2015;11(2):e1004662 10.1371/journal.ppat.1004662 ; PubMed Central PMCID: PMC4339193. 25710374 PMC4339193

[76] Lee HP, Jun YC, Choi JK, Kim JI, Carp RI, Kim YS. Activation of mitogen-activated protein kinases in hamster brains infected with 263K scrapie agent. J Neurochem. 2005;95(2):584–93. Epub 2005/09/02. 10.1111/j.1471-4159.2005.03429.x . 16135077

[77] Puig B, Altmeppen HC, Ulbrich S, Linsenmeier L, Krasemann S, Chakroun K, et al Secretory pathway retention of mutant prion protein induces p38-MAPK activation and lethal disease in mice. Sci Rep. 2016;6:24970 Epub 2016/04/28. 10.1038/srep24970 ; PubMed Central PMCID: PMCPMC4847012. 27117504 PMC4847012

[78] Villa V, Corsaro A, Thellung S, Paludi D, Chiovitti K, Venezia V, et al Characterization of the proapoptotic intracellular mechanisms induced by a toxic conformer of the recombinant human prion protein fragment 90–231. Ann NY Acad Sci. 2006;1090:276–91. Epub 2007/03/27. 10.1196/annals.1378.030 . 17384271

[79] Pietri M, Dakowski C, Hannaoui S, Alleaume-Butaux A, Hernandez-Rapp J, Ragagnin A, et al PDK1 decreases TACE-mediated α-secretase activity and promotes disease progression in prion and Alzheimer's diseases. Nat Med. 2013;19(9):1124–31. Epub 2013/08/21. 10.1038/nm.3302 . 23955714

[80] Goniotaki D, Lakkaraju AKK, Shrivastava AN, Bakirci P, Sorce S, Senatore A, et al Inhibition of group-I metabotropic glutamate receptors protects against prion toxicity. PLoS Pathog. 2017;13(11):e1006733 Epub 2017/11/28. 10.1371/journal.ppat.1006733 . 29176838 PMC5720820

[81] Müller WEG, Ushijima H, Schroder HC, Forrest JM, Schatton WF, Rytik PG, et al Cytoprotective effect of NMDA receptor antagonists on prion protein (Prion^Sc^)-induced toxicity in rat cortical cell cultures. Eur J Pharmacol. 1993;246(3):261–7. . 7901042 10.1016/0922-4106(93)90040-g

[82] Korte S, Vassallo N, Kramer ML, Kretzschmar HA, Herms J. Modulation of L-type voltage-gated calcium channels by recombinant prion protein. J Neurochem. 2003;87(4):1037–42. Epub 2003/11/19. . 14622132 10.1046/j.1471-4159.2003.02080.x

[83] Trevitt CR, Collinge J. A systematic review of prion therapeutics in experimental models. Brain. 2006;129(Pt 9):2241–65. 10.1093/brain/awl150 . 16816391

[84] Orru CD, Bongianni M, Tonoli G, Ferrari S, Hughson AG, Groveman BR, et al A test for Creutzfeldt-Jakob disease using nasal brushings. N Engl J Med. 2014;371(6):519–29. 10.1056/NEJMoa1315200 ; PubMed Central PMCID: PMC4186748. 25099576 PMC4186748

[85] Moda F, Gambetti P, Notari S, Concha-Marambio L, Catania M, Park KW, et al Prions in the urine of patients with variant Creutzfeldt-Jakob disease. N Engl J Med. 2014;371(6):530–9. 10.1056/NEJMoa1404401 ; PubMed Central PMCID: PMC4162740. 25099577 PMC4162740

[86] Howard R, McShane R, Lindesay J, Ritchie C, Baldwin A, Barber R, et al Donepezil and memantine for moderate-to-severe Alzheimer's disease. N Engl J Med. 2012;366(10):893–903. Epub 2012/03/09. 10.1056/NEJMoa1106668 . 22397651

[87] Coulthard LR, White DE, Jones DL, McDermott MF, Burchill SA. p38^MAPK^: stress responses from molecular mechanisms to therapeutics. Trends Mol Med. 2009;15(8):369–79. Epub 2009/08/12. 10.1016/j.molmed.2009.06.005 ; PubMed Central PMCID: PMCPMC3016890. 19665431 PMC3016890

[88] Borders AS, de Almeida L, Van Eldik LJ, Watterson DM. The p38α mitogen-activated protein kinase as a central nervous system drug discovery target. BMC Neurosci. 2008;9 Suppl 2:S12 Epub 2009/01/06. 10.1186/1471-2202-9-S2-S12 ; PubMed Central PMCID: PMCPMC2604896. 19090985 PMC2604896

[89] Yasuda S, Sugiura H, Tanaka H, Takigami S, Yamagata K. p38 MAP kinase inhibitors as potential therapeutic drugs for neural diseases. Cent Nerv Syst Agents Med Chem. 2011;11(1):45–59. Epub 2010/09/04. . 20812905 10.2174/187152411794961040

[90] Munoz L, Ammit AJ. Targeting p38 MAPK pathway for the treatment of Alzheimer's disease. Neuropharmacology. 2010;58(3):561–8. Epub 2009/12/03. 10.1016/j.neuropharm.2009.11.010 . 19951717

[91] Jucker M, Walker LC. Self-propagation of pathogenic protein aggregates in neurodegenerative diseases. Nature. 2013;501(7465):45–51. 10.1038/nature12481 . 24005412 PMC3963807

[92] Ferreira DG, Temido-Ferreira M, Miranda HV, Batalha VL, Coelho JE, Szego EM, et al α-synuclein interacts with PrP^C^ to induce cognitive impairment through mGluR5 and NMDAR2B. Nat Neurosci. 2017;20(11):1569–79. Epub 2017/09/26. 10.1038/nn.4648 . 28945221

[93] Kaech S, Banker G. Culturing hippocampal neurons. Nat Protoc. 2006;1(5):2406–15. 10.1038/nprot.2006.356 . 17406484

[94] Miranda CJ, Braun L, Jiang Y, Hester ME, Zhang L, Riolo M, et al Aging brain microenvironment decreases hippocampal neurogenesis through Wnt-mediated survivin signaling. Aging cell. 2012;11(3):542–52. 10.1111/j.1474-9726.2012.00816.x ; PubMed Central PMCID: PMC3350615. 22404871 PMC3350615

[95] Srivastava DP, Woolfrey KM, Penzes P. Analysis of dendritic spine morphology in cultured CNS neurons. J Vis Exp. 2011;(53):e2794 10.3791/2794 ; PubMed Central PMCID: PMCPMC3196192. 21775964 PMC3196192

[96] Shen W, Wu B, Zhang Z, Dou Y, Rao ZR, Chen YR, et al Activity-induced rapid synaptic maturation mediated by presynaptic cdc42 signaling. Neuron. 2006;50(3):401–14. Epub 2006/05/06. 10.1016/j.neuron.2006.03.017 . 16675395

[97] Biasini E, Medrano AZ, Thellung S, Chiesa R, Harris DA. Multiple biochemical similarities between infectious and non-infectious aggregates of a prion protein carrying an octapeptide insertion. J Neurochem. 2008;104:1293–308. 10.1111/j.1471-4159.2007.05082.x 18034781

[98] Turnbaugh JA, Unterberger U, Saa P, Massignan T, Fluharty BR, Bowman FP, et al The N-terminal, polybasic region of PrP^C^ dictates the efficiency of prion propagation by binding to PrP^Sc^. J Neurosci. 2012;32(26):8817–30. Epub 2012/06/30. doi: 32/26/8817 [pii] 10.1523/JNEUROSCI.1103-12.2012 ; PubMed Central PMCID: PMC3433751. 22745483 PMC3433751

[99] Safar JG, Scott M, Monaghan J, Deering C, Didorenko S, Vergara J, et al Measuring prions causing bovine spongiform encephalopathy or chronic wasting disease by immunoassays and transgenic mice. Nat Biotechnol. 2002;20(11):1147–50. 10.1038/nbt748 . 12389035

[100] Klein WL. Aβ toxicity in Alzheimer's disease: globular oligomers (ADDLs) as new vaccine and drug targets. Neurochem Int. 2002;41(5):345–52. . 12176077 10.1016/s0197-0186(02)00050-5

