# Supplementary figures and images for: Prions activate a p38 MAPK synaptotoxic signaling pathway
Source: PLoS Pathog. 2018 Sep 20;14(9):e1007283. doi: 10.1371/journal.ppat.1007283 (PMC6147624; doi:10.1371/journal.ppat.1007283)

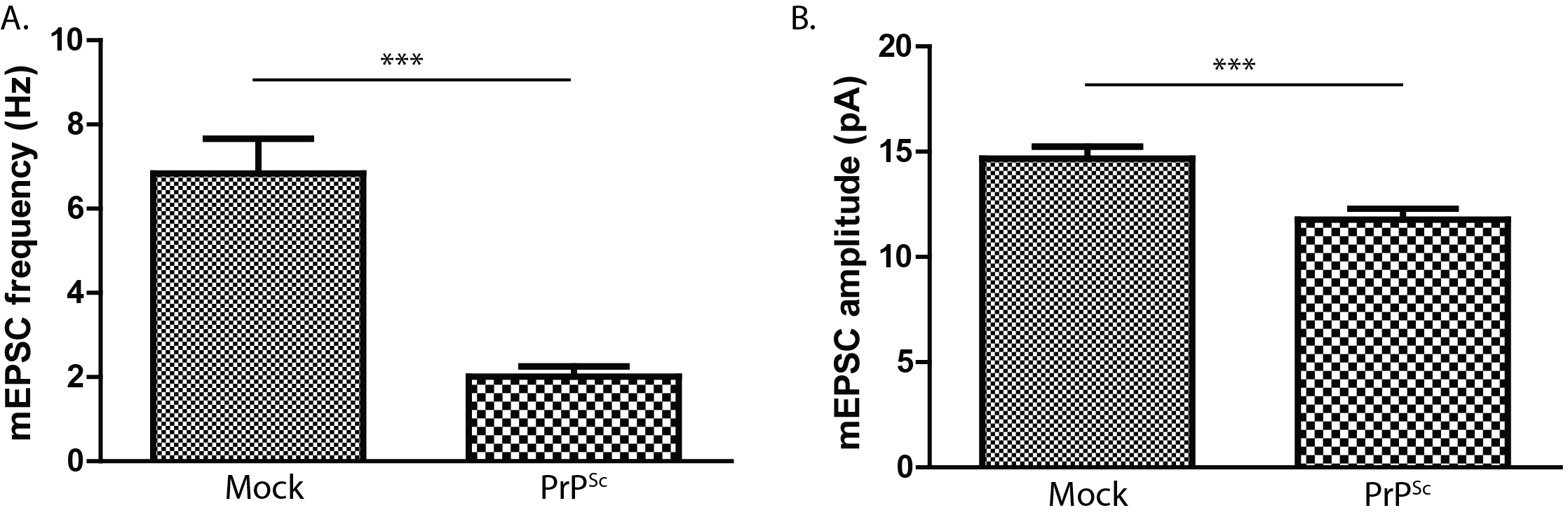

Supplement: S1 Fig — Hippocampal neurons were cultured on coverslips at low-density over an astrocyte feeder layer, the same procedure used for visualization of dendritic spines. Cultures were treated for 24 hrs with either purified PrPSc or with mock-purified material, after which mEPSCs were recorded and their frequencies (A) and amplitudes (B) quantitated. N = 15 from 2 independent experiments. ***p<0.001 by Student’s t-test. (TIF) [file ppat.1007283.s001.tif]

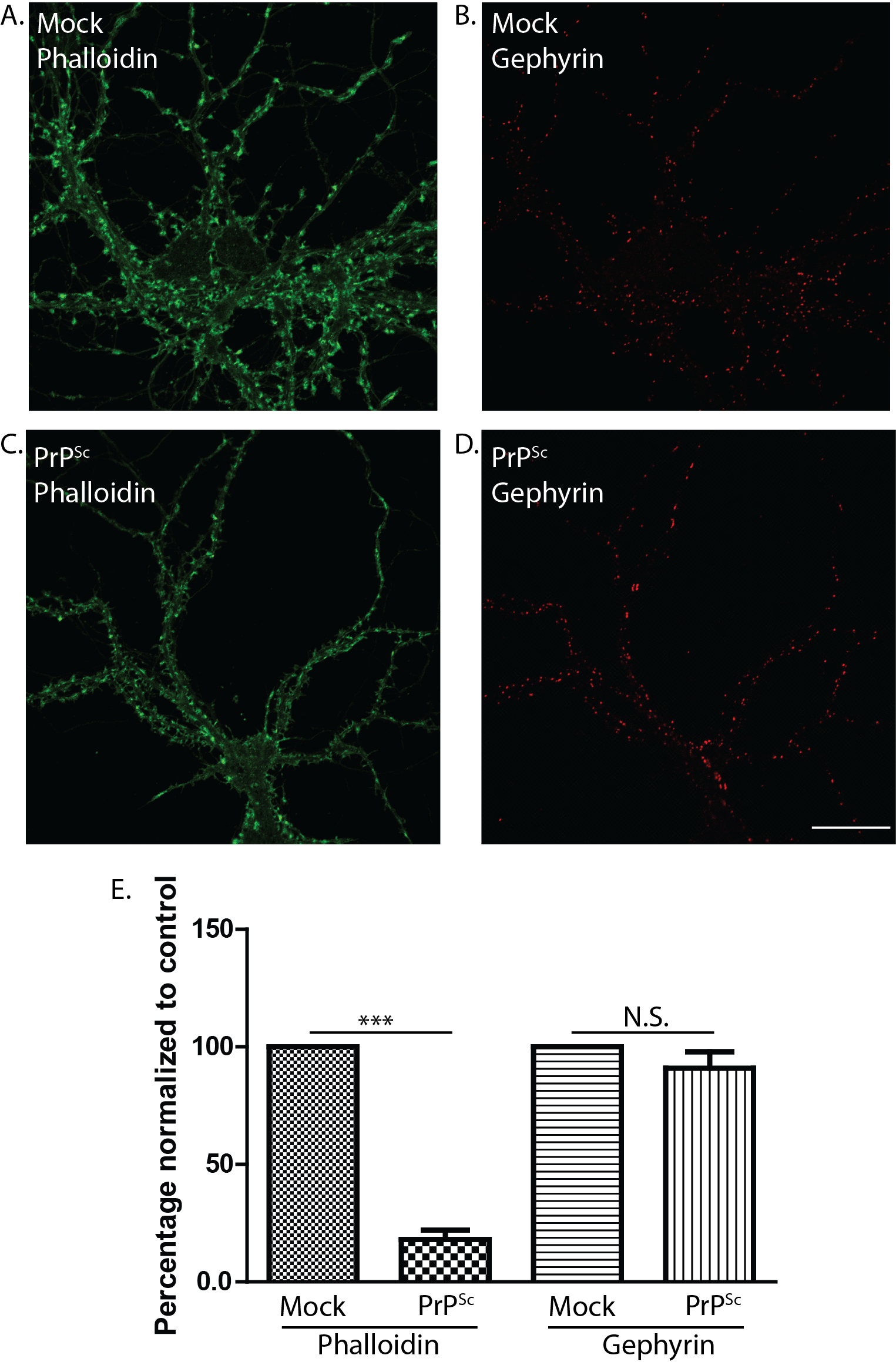

Supplement: S2 Fig — Hippocampal neurons were treated for 24 hrs with mock-purified material (A, B) or with purified PrPSc (C, D). Cultures were then fixed and stained with fluorescent phalloidin (green) (A, C) along with an antibody to the inhibitory postsynaptic marker, gephyrin (red) (B, D). Quantitation of spine number and gephyrin staining is shown in panel E, normalized to the values in mock-treated cultures. Pooled measurements were collected from 15–20 cells from 3 independent experiments. ***p<0.001 by Student’s t-test; N.S., not significantly different. Scale bar in panel D = 20 μm (also applicable to panels A-C). (TIF) [file ppat.1007283.s002.tif]

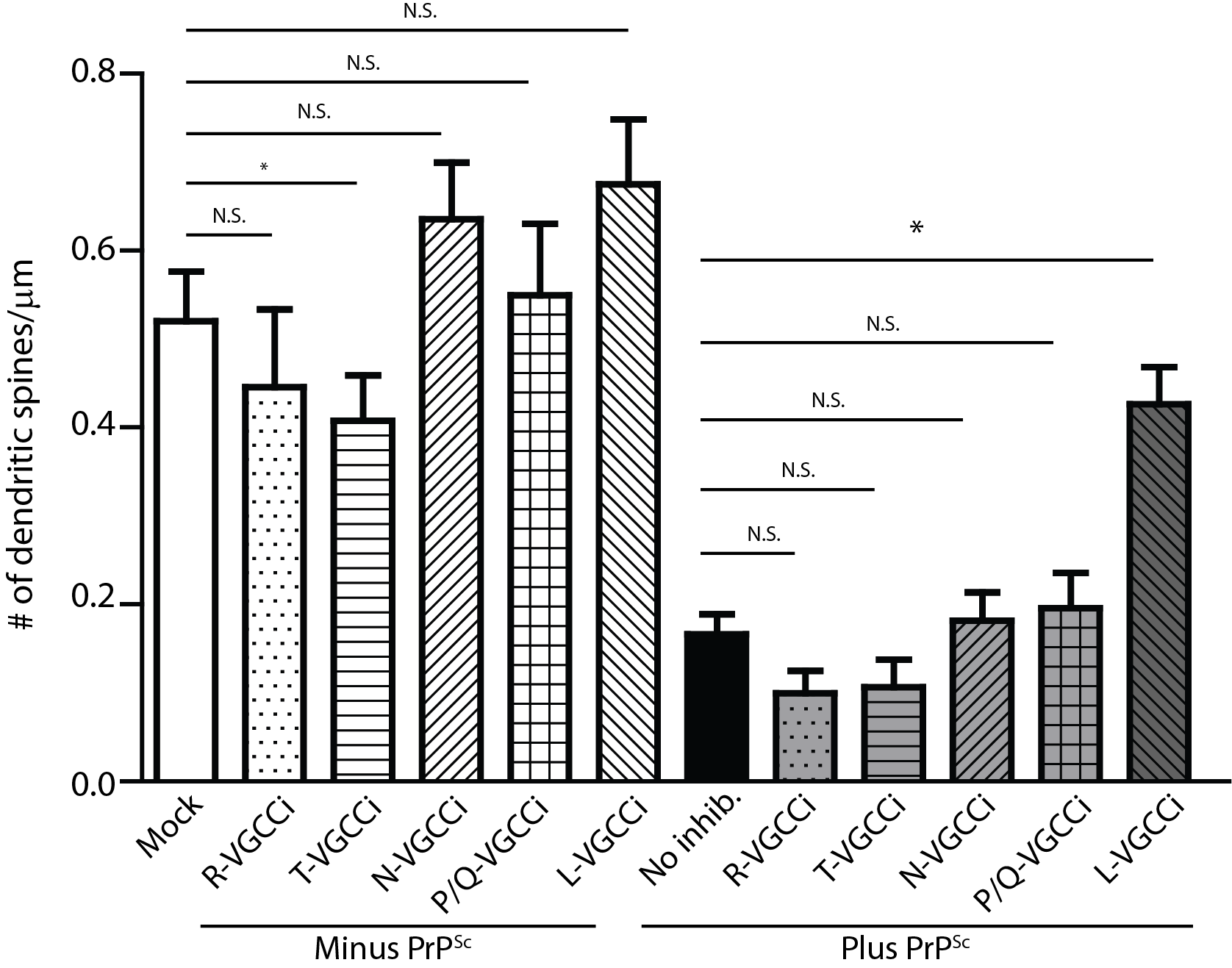

Supplement: S3 Fig — Hippocampal neurons were treated for 24 hrs with purified PrPSc in the presence or absence of inhibitors of R-, T-, N-, P/Q- and L-type voltage-gated calcium channels (VGCCs) (bars labeled Plus PrPSc). A parallel set of cultures was treated with inhibitor without PrPSc (bars labeled Minus PrPSc). The bar labeled Mock represents cultures treated with mock-purified material in the absence of inhibitors. Pooled measurements of spine number were collected from 15–20 cells from 3 independent experiments. *p<0.05; ***p<0.001 by Student’s t-test; N.S., not significantly different. The inhibitors used are listed in Table 1. (TIF) [file ppat.1007283.s003.tif]

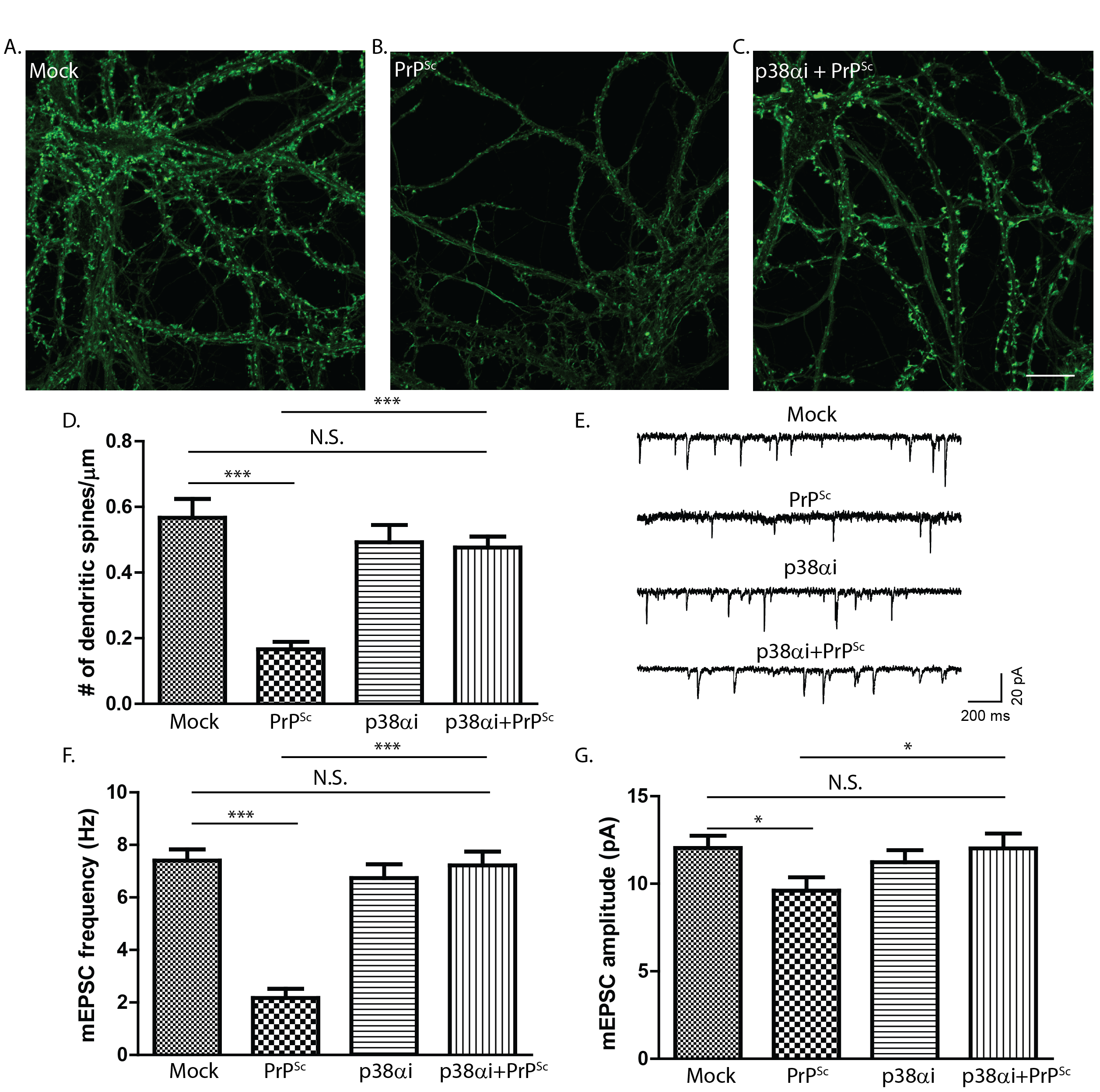

Supplement: S4 Fig — Hippocampal neurons were treated for 24 hrs with mock-purified material (A), purified PrPSc (B), or purified PrPSc in the presence of a p38α MAPK inhibitor (VX745, 100 nM) (C). Dendritic spines were then visualized by fluorescent phalloidin staining (A-C). Pooled measurements of spine number were collected from 15–20 cells from 3 independent experiments (D). The bar labeled p38αi represents cultures treated with inhibitor without PrPSc. Parallel cultures were analyzed by patch clamping to measure mEPSC frequency and amplitude (E-G).). N = 10 cells from 2 independent experiments. ***p<0.001 and * p<0.05 by Student’s t-test; N.S., not significantly different. Scale bar in panel C = 20 μm (also applicable to panels A and B). (TIF) [file ppat.1007283.s004.tif]

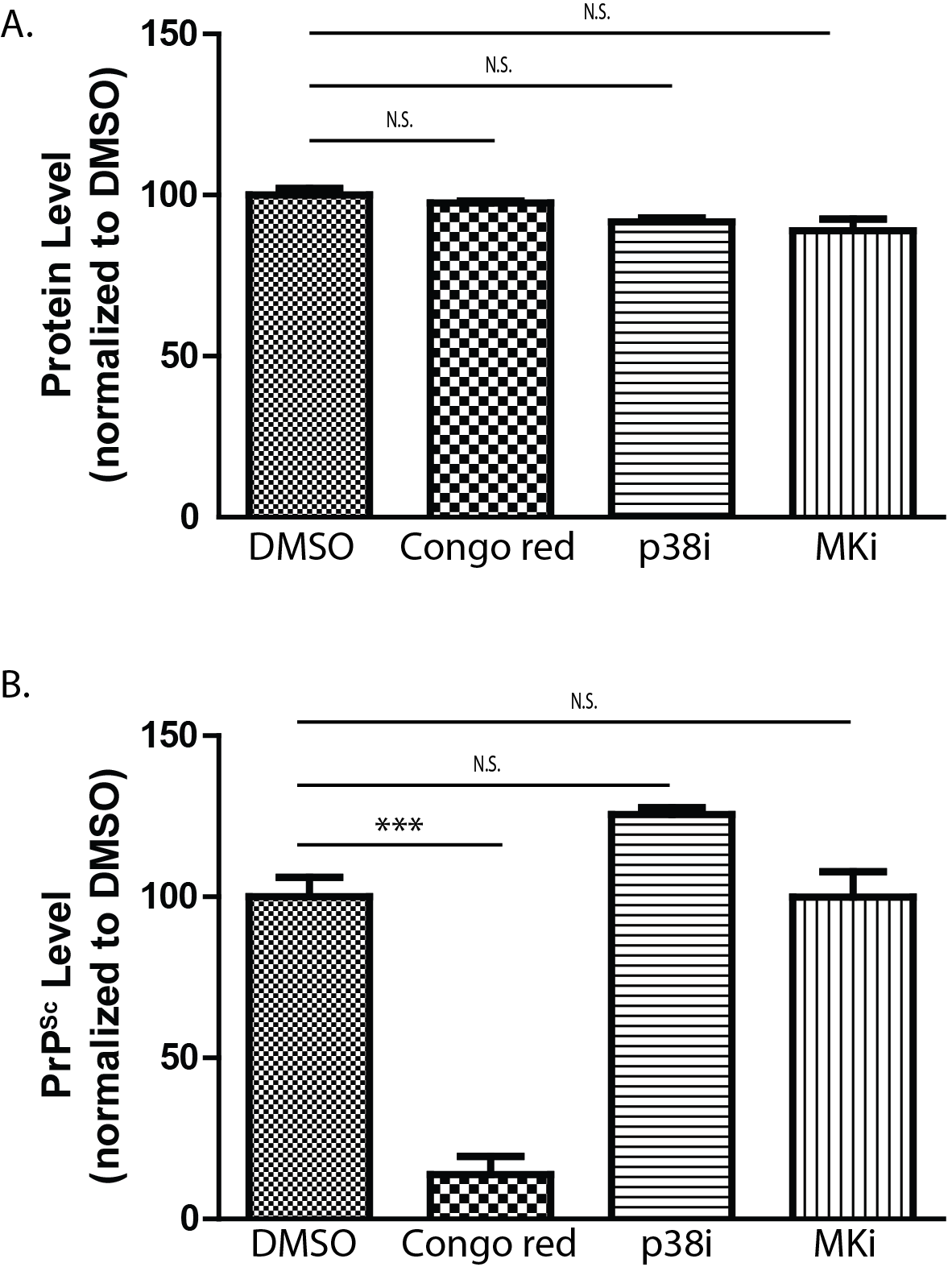

Supplement: S5 Fig — ScN2a cells were treated for 3 days with DMSO vehicle, Congo red (5 μm), p38 MAPK inhibitor (SB239063, 10 μM), or MK2/3/5 inhibitor (CAS1186648, 500 nM), after which cells were split at a 1:5 ratio and fresh inhibitors were added for 4 more days. At the end of the 7-day treatment, cells were harvested and lysed. BCA protein assays of lysates were performed as a measure of drug cytotoxicity (A). Cell lysates were also subjected to proteinase K digestion followed by Western blotting to reveal proteinase K-resistant PrPSc (B). ***p<0.001 by Student’s t-test; N.S., not significantly different. Data were derived from triplicate cultures. (TIF) [file ppat.1007283.s005.tif]

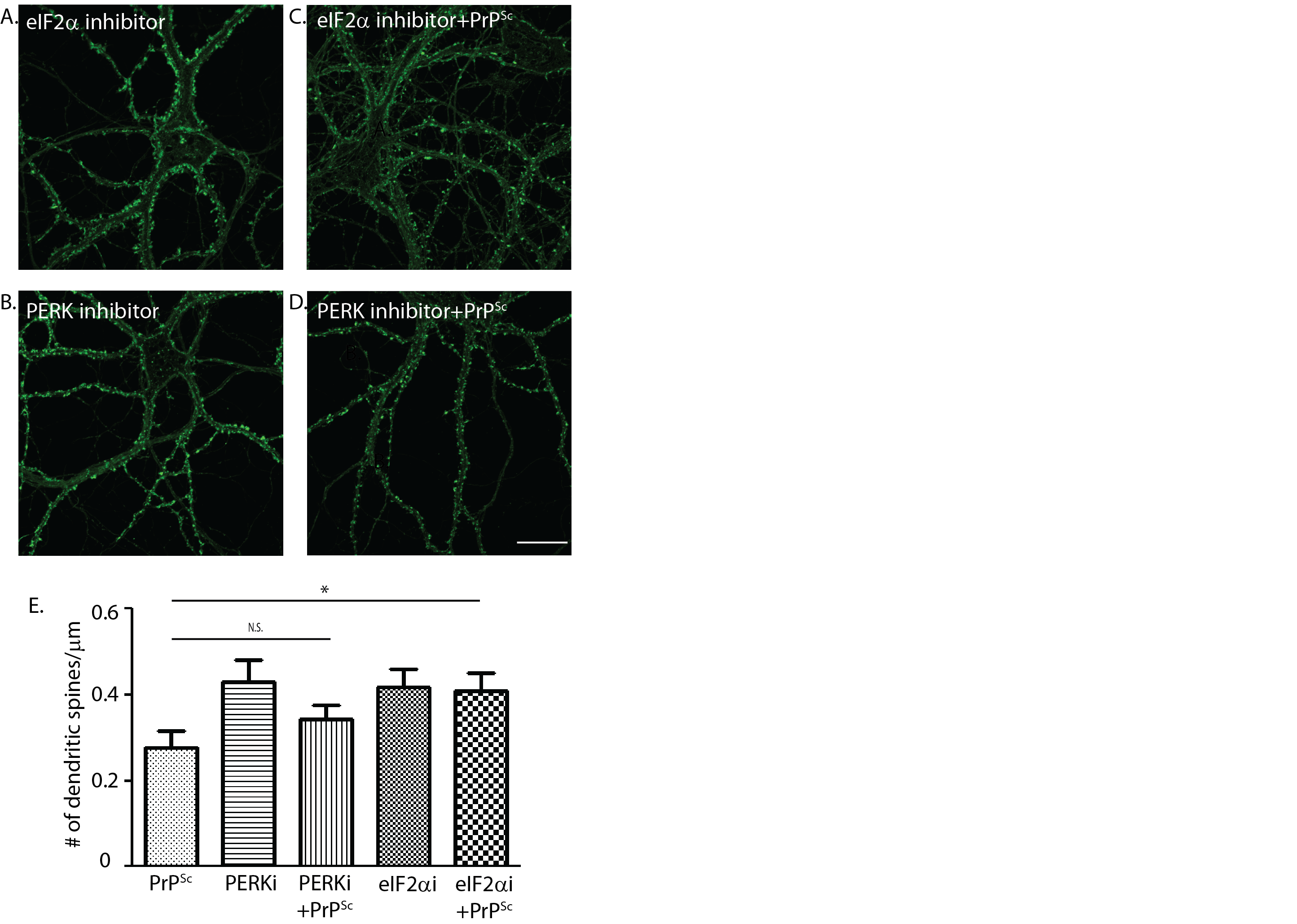

Supplement: S6 Fig — Hippocampal neurons from WT mice were treated for 24 hr with integrated stress response inhibitor (Trans-ISRIB, 20 nM) alone (A), PERK inhibitor (GSK2606414, 500 nM) alone (B), or with the respective inhibitors in combination with purified PrPSc (C, D). Neurons were then fixed and stained with fluorescent phalloidin. Pooled measurements of dendritic spine number were collected from 15–20 cells from 3 independent experiments (E). *p<0.05 by Student’s t-test; N.S., not significantly different. Scale bar in panel D = 20 μm (also applicable to panels A-C). (TIF) [file ppat.1007283.s006.tif]

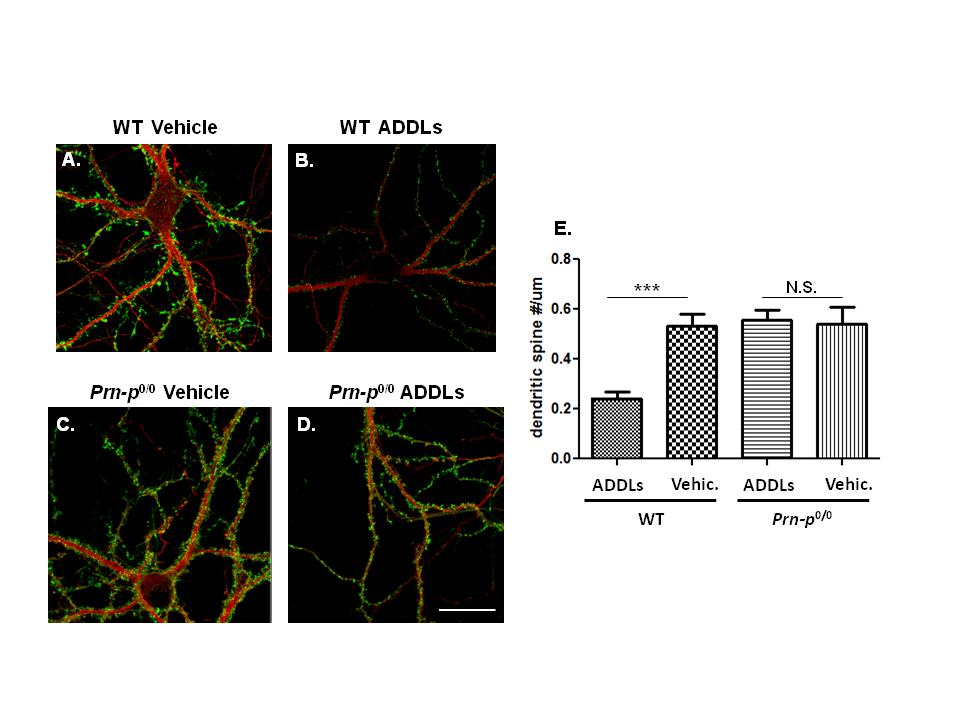

Supplement: S7 Fig — Primary hippocampal neurons from wild-type (WT) mice (A, B) or PrP knockout mice (Prn-p0/0) (C, D) were treated for 24 hrs with vehicle (A, C) or ADDLs (1.6 μM) (B, D). Neurons were then fixed and stained with fluorescent phalloidin (green) to visualize F-actin in dendritic spines, and with anti-tubulin (red) to visualize overall dendritic morphology. Pooled measurements of dendritic spine number were collected from 15–20 cells from 3 indepdendent experiments (E). ***p<0.001 by Student’s t-test; N.S., not significantly different. Scale bar in panel D = 20 μm (also applicable to panels A-C). (TIF) [file ppat.1007283.s007.tif]
